# Transcriptome Analysis of Flower Sex Differentiation in *Jatropha curcas* L. Using RNA Sequencing

**DOI:** 10.1371/journal.pone.0145613

**Published:** 2016-02-05

**Authors:** Gang Xu, Jian Huang, Yong Yang, Yin-an Yao

**Affiliations:** 1 Research Institute of Forest Resources and Environment, Guizhou University, Guiyang, Guizhou, P. R. China; 2 Key Laboratory of Green Pesticide and Agricultural Bioengineering, Ministry of Education, Center for Research and Development of Fine Chemicals of Guizhou University, Guiyang, Guizhou, P. R. China; 3 School of life science and engineering, Southwest University of Science and Technology, Mianyang, Sichuan, P. R. China; 4 Institute of Entomology, Guizhou University, Guiyang, Guizhou, P. R. China; Wuhan University, CHINA

## Abstract

**Background:**

*Jatropha curcas* is thought to be a promising biofuel material, but its yield is restricted by a low ratio of instaminate / staminate flowers (1/10-1/30). Furthermore, valuable information about flower sex differentiation in this plant is scarce. To explore the mechanism of this process in *J*. *curcas*, transcriptome profiling of flower development was carried out, and certain genes related with sex differentiation were obtained through digital gene expression analysis of flower buds from different phases of floral development.

**Results:**

After Illumina sequencing and clustering, 57,962 unigenes were identified. A total of 47,423 unigenes were annotated, with 85 being related to carpel and stamen differentiation, 126 involved in carpel and stamen development, and 592 functioning in the later development stage for the maturation of staminate or instaminate flowers. Annotation of these genes provided comprehensive information regarding the sex differentiation of flowers, including the signaling system, hormone biosynthesis and regulation, transcription regulation and ubiquitin-mediated proteolysis. A further expression pattern analysis of 15 sex-related genes using quantitative real-time PCR revealed that gibberellin-regulated protein 4-like protein and AMP-activated protein kinase are associated with stamen differentiation, whereas auxin response factor 6-like protein, AGAMOUS-like 20 protein, CLAVATA1, RING-H2 finger protein ATL3J, auxin-induced protein 22D, and r2r3-myb transcription factor contribute to embryo sac development in the instaminate flower. Cytokinin oxidase, Unigene28, auxin repressed-like protein ARP1, gibberellin receptor protein GID1 and auxin-induced protein X10A are involved in both stages mentioned above. In addition to its function in the differentiation and development of the stamens, the gibberellin signaling pathway also functions in embryo sac development for the instaminate flower. The auxin signaling pathway also participates in both stamen development and embryo sac development.

**Conclusions:**

Our transcriptome data provide a comprehensive gene expression profile for flower sex differentiation in *Jatropha curcas*, as well as new clues and information for further study in this field.

## Introduction

*Jatropha curcas* L., which belongs to the family *Euphorbiaceae*, is endemic to tropical America and is now prevalent in Africa and Asia. Being composed of 75% unsaturated fatty acids, the oil from *J*. *curcas* seeds is suitable for producing biodiesel. In addition to biodiesel, *J*. *curcas* has potential use in the production of medicinal compounds. Moreover, due to its low nutrient requirement and drought tolerance, *J*. *curcas* can grow on wastelands, degraded lands and mine-contaminated lands, and afforestation with this plant has the potential to meet the increasing demand for oil seed crops without sacrificing agricultural land. However, in many locations, the planting of *J*. *curcas* brings little economic benefit due to the low seed yield of this plant, which greatly prevents the exploitation and expansion of this energy crop. Indeed, the yield in semi-arid areas or wasteland is 2–3 ton ha^-1^ yr^-1^ [1–3]. Even under conditions that are suitable for its growth (good soil and an average annual rainfall of 900–1200 mm), the dry seed yield under optimal management practice is only 5 ton ha^-1^ yr^-1^ [2–4]. As the small ratio of instaminate to staminate flowers (1/10–1/30) is one of the critically limited factors for the low seed yield of *J*. *curcas* [5], exploring the mechanism of floral sex differentiation in this plant is urgent; this valuable information would provide clues for improving seed yield.

As a new biofuel plant, *J*. *curcas* has attracted much attention of researchers, and studies on its genetics and genomics have been conducted in recent years. The genome size of *J*. *curcas* is estimated at 410 Mb [6]. Natarajan et al. [7] obtained 12,084 expressed sequence tags (ESTs) from developing seeds, and the contig assembly of these ESTs resulted in 6,361 unigenes. In the study of Costa et al. [8], 13,249 ESTs were obtained from developing and germinating endosperm and were assembled into 4,622 unigenes. Wang et al. [9] reported the gene expression profiles of the flower bud at the metaphase stage and of the developing seed from the early to metaphase stages after pollination; a total of 9,289 ESTs were obtained and then assembled into 4,502 unique sequences. Ban et al (2014) analyzed the transcriptome of the inflorescence meristems of *Jatropha curcas* treated with cytokinin, obtaining 81,736 unigenes, providing comprehensive information of cytokinin- response genes [10]. A total of 40,929 complete and partial sequences of protein-encoding genes were obtained by Sato et al. [11] through genome sequence analysis. Several flower development-related genes have also been identified, including a flowering time regulator (SVP), three floral meristem identity genes (APETALA2, APETALA3, and PISTILLATA), and five flowering regulators (CONSTANS, FLOWERING LOCUS D and F, LEAFY, and SUPPRESSOR OF OVEREXPRESSION OF CONSTANS1). However, molecular information about the florescence processes of *J*. *curcas*, including the gene expression profiles of different flower development stages, the genes involved in flower development, and sex-related genes, is still very limited.

In this research, Illumina short-read technology and a digital gene expression (DGE) system were used, and *de novo* transcriptome assembly and sequence annotation were performed. DGE libraries from flower buds at different stages for sex differentiation and floral development were compared with each other, which would help in identifying differentially expressed genes (DEGs) that are involved in the sex differentiation of flowers and in gaining insight into the mechanism of flower sex differentiation in *J*. *curcas*.

## Results

### Morphological observation of developing flowers

**Fig 1:**
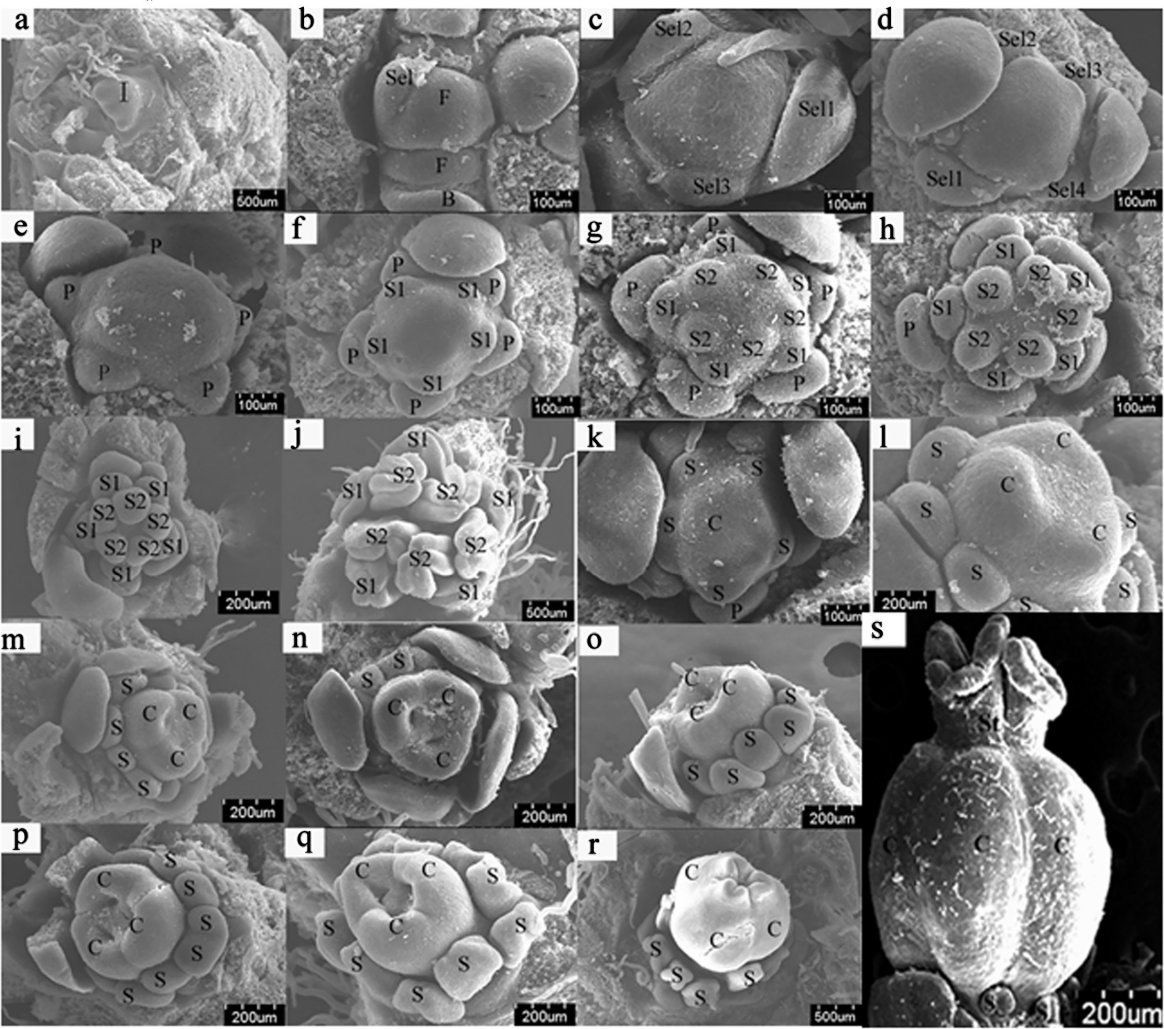
SEM photographs (a-s) of *Jatropha curcas* floral organogenesis. SEM photographs of *Jatropha curcas* floral organogenesis. **a.** Inflorescence primordium. **b.** Two floral primordia of different sizes initiate in the center of the inflorescence primordium. The first sepal primordium initiates. **c**. Three sepals of different sizes initiate. **d.** Five sepals of different sizes initiate. **e.** Five petal primordia initiate sequentially. **f.** The initiation of the outer whorl of stamen primordia. **g.** The initiation of the inner whorl of stamen primordia. **h.** Stamens of the outer whorl develop faster than those of the inner whorl. **i.** The developing ten stamens. **j.** The mature anthers are situated in the abaxial surface (outward anther). **k.** Carpel primordia initiate. **l.** Distinct carpel. **m-p.** Stamens and carpels continue to develop. **q.** Carpels close gradually, and stamens stop developing. **r.** In the instaminate flower, the ovary bulges, and the stamens become smaller and degenerate. **s.** The stamens had degenerated, and the stigma was formed. B, Bracts and bract primordia; C, carpels and carpel primordia; F, floral primordia; I, inflorescence primordia; P, petals and petal primordia; S1, the outer whorl of stamens; S2, the inner whorl of stamens; Se1~Se5, the first sepal, the second sepal, …, the fifth sepal (according to the order of initiation), respectively; St: stigma. Stage 1 (S1): from a to e; stage 2 (S2): from f to h; stage 3 (S3): from i to j; stage 4 (S4): k-l; stage 5 (S5): from m to p; stage 6 (S6): from q to s (S6-1: q, the carpels began to fuse; S6-2: r, the carpels were fused; S6-3: s, the stigma was formed.) (Fig 1s is from “Liu H, Deng Y, Liao J (2008) Floral organogenesis of three species of *Jatropha* (Euphorbiaceae). Journal of Systematics and Evolution 46 (1): 53–61.”).

Sex differentiation occurred after the initiation of five petal primordia; unlike staminate flower, the instaminate flower experienced a hermaphrodite period. During the development of staminate flowers, the five outer whorls of stamen primordia were initiated first, followed by the five inner whorls of stamen primordia. The instaminate flowers experienced a period of bisexual flowers. When the three carpel primordia were initiated, ten stamen primordia were also initiated and developed along with the development of the carpels. As the three carpels began to fuse, the development of the stamens was arrested and gradually degenerated as the ovary bulged (Fig 1). The sex differentiation process of *J*. *curcas* flowers could be divided into six stages: stage 1: sex differentiation is not initiated; stage 2: from stamen primordia beginning to differentiate to ten stamen primordia formed; stage 3: from ten stamen primordia formed to mature staminate flowers; stage 4: from carpel primordia beginning to differentiate to three distinct carpels formed; stage 5: carpels continue developing; stage 6: from carpels beginning to fuse to mature instaminate flowers (Fig 1).

Using an anatomical lens, the external morphology of flower organs, especially the stamens and the carpels, were observed to identify the development stage of flower buds. The flower bud samples were then divided into six groups according to their development stages: sample 1 (S1), the flower buds of stage 1; sample 2 (S2), the flower buds of stage 2; sample 3 (S3), the flower buds of stage 3; sample 4 (S4), the flower buds of stage 4; sample 5 (S5), the flower buds of stage 5; and sample 6 (S6), the flower buds of stage 6 (Fig 1).

### Illumina transcriptome sequencing and assembly of clean reads

**Fig 2:**
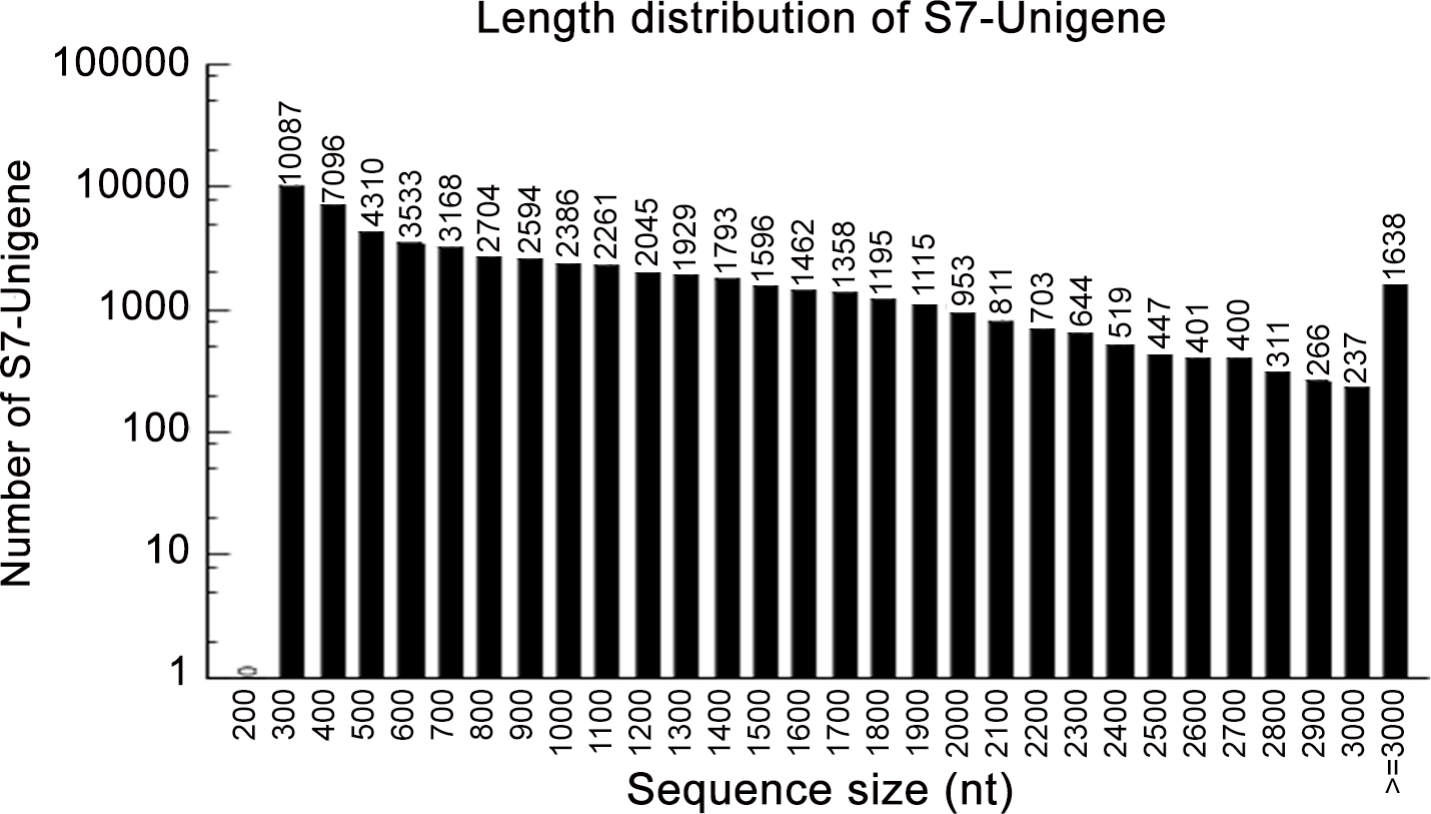
Length distribution of the unigenes from the sample.

**Fig 3:**
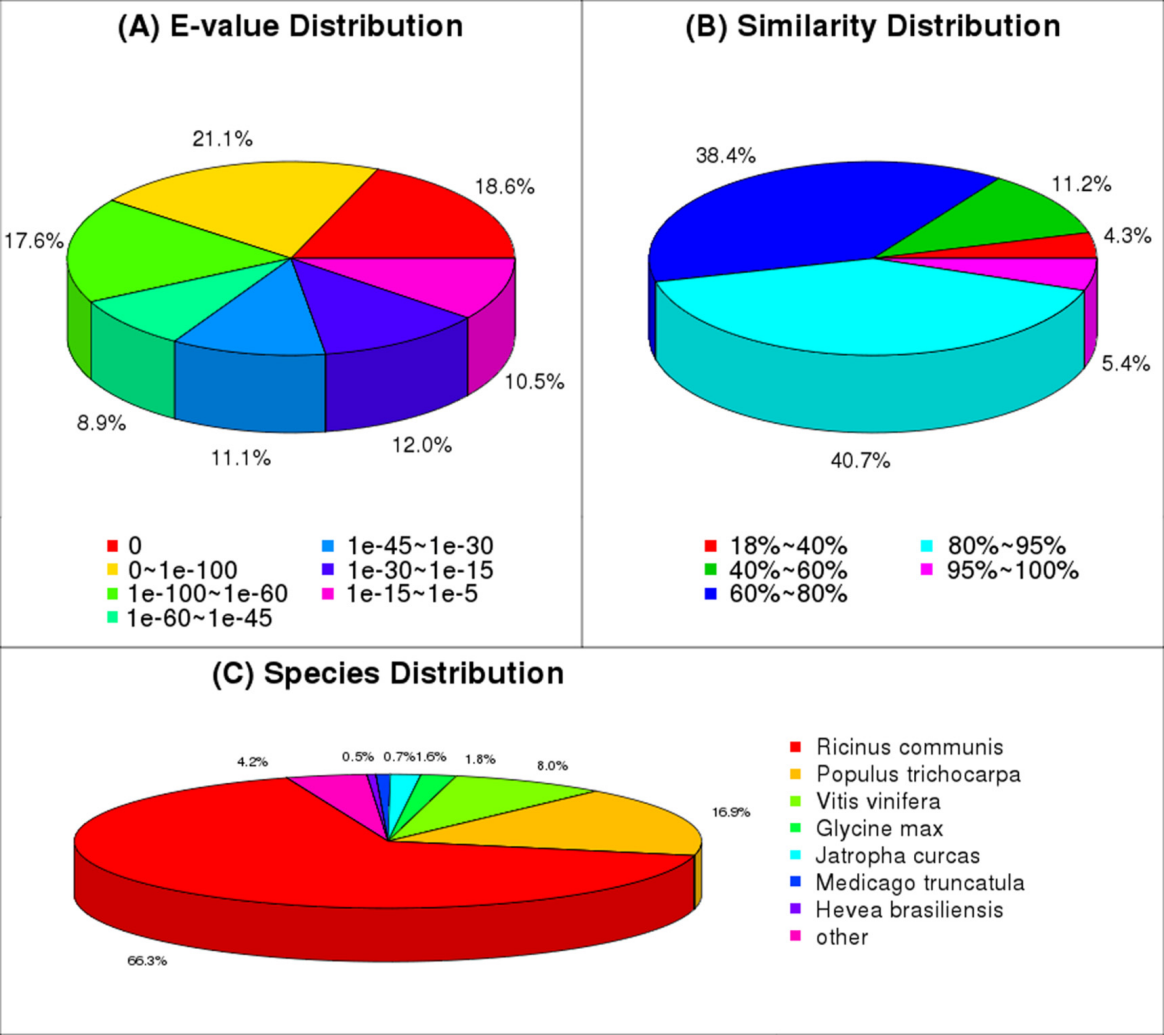
Data of NR classification. (A) E-value distribution of BLAST hits for each unique sequence with a cut-off E-value of 1.0E^-5^. (B) Similarity distribution of the top BLAST hits for each sequence. (C) The species distribution is shown as a percentage of the total homologous sequences, with an E-value of at least 1.0E^-5^.

To analyze the transcriptome of *J*. *curcas*, mRNA was isolated from total RNA and then fragmented; these mRNA fragments were used as templates to synthesize cDNA. The obtained cDNA was sequenced using the Illumina sequencing platform. After filtering the adaptors and low-quality sequences, 54,996,594 clean reads with a mean length of 90 bp were obtained (Table 1). These clean reads were then assembled using short reads assembling software (Trinity). As a result, a total of 57,962 unigenes were obtained, including 26,689 clusters and 31,273 singletons (Table 2), with a mean length of 988 bp and an N50 of 1474 (i.e., 50% of the assembled bases were assembled into a unigene with a length of 1474 bp or longer) (Table 2, Fig 2). The E-value and similarity distribution of the best hits against the nr database were shown in Fig 3A and 3B, respectively.

**Table 1:** Statistics of sequence output.

Sample	Total row reads	Total clean reads	Total clean nucleotides (nt)	Q20 percentage	N percentage	GC percentage
S7	60,723,596	54,996,594	4,949,693,460	96.78%	0.00%	44.25%

**Table 2:** Statistics of assembly quality.

	Sample	Total Number	Total Length(nt)	Mean Length (nt)	N50	Total Consensus Sequences	Distinct Clusters	Distinct Singletons
Contig	S7	102,074	39,160,389	384	904	-	-	-
Unigene	S7	57,962	57,265,503	988	1474	57,962	26,689	31,273

### Annotation of *J*. *curcas* transcripts

For unigene annotation, the obtained 57,962 unigenes were searched using BLASTx against the NR, NT, Swiss-Prot, KEGG, COG and GO databases. A total of 47,423 unigenes returning significant BLAST hits (E-value<1.0E^-5^) were annotated, including 45,565 in NR, 44,966 in NT, 27,739 in Swiss-Prot, 26,600 in KEGG, 17,731 in COG, and 39,143 in GO (Table 3). With regard to species distribution, the highest percentage of unique sequences matched to the genes of *Ricinus communis* (66.3%), followed by *Populus trichocarpa* (16.9%), *Vitis vinifera* (8.0%), *Glycine max* (1.8%), *J*. *curcas* (1.6%), *Medicago truncatula* (0.7%), *Hevea brasiliensis* (0.5%) and other unclear proteins (4.2%) (Fig 3C). It is possible that only a few unigenes matched the EST database because the final *J*. *curcas* genome data are not yet available in NCBI.

**Table 3:** Summary of annotation results.

Sample	NR	NT	Swiss-Prot	KEGG	COG	GO	ALL
S7	45,565	44,966	27739	26600	17731	39143	47423

### Sequencing of DGE libraries and alignment with a reference database

**Fig 4:**
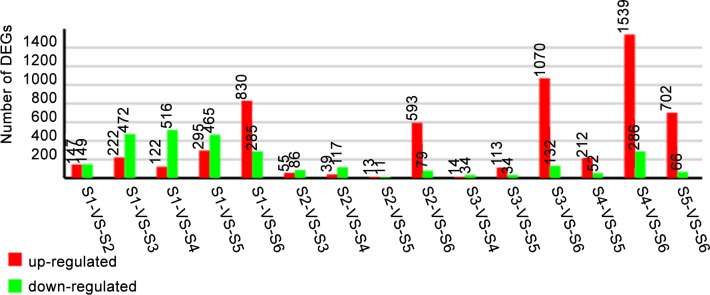
The results of a comparison of the gene expression level between different samples. Expression comparisons were performed between S1 and S2, S1 and S3, S1 and S4, S1 and S5, and S1 and S6.

Eighteen DGE libraries for the samples at different flower development phases were sequenced. The analysis for the sequencing saturation, homogenization and randomness suggested that these results could reflect the actual expression of genes; the data were thus suitable for further analysis (S1 Fig). The correlation test for the gene expression levels between the biological replicates (R^2^>0.92) showed that the expression patterns in our experiments reflected the actual expression of genes for the samples at different floral developmental stages (S2 Fig). The differentially expressed genes (DEGs) identified in the different development phases are shown in Fig 4.

### Predicted genes involved in floral development and sex differentiation

Regarding the DEGs related to the transition of the meristem from vegetative to reproductive phase, transcript abundance for the majority of genes was reduced in the maturation stage of stamen (S1-S2-S3) or in the early development stage of instaminate flower (S1-S4), but enhanced in S6 of instaminate flower development (S3 Fig). Transcripts for the S-locus-specific-glycoprotein-S6-precursor (unigene26458_S7), F-box and WD40-domain protein (Unigene25463_S7), S-adenosylmethionine_decarboxylase (CL2711.Contig3_S7), and flowering-related B-class MADS-box protein (Unigene20469_S7) genes were increased in the mature stage in both staminate (S1-S2-S3) and instaminate organs (S1-S4-S5-S6). In contrast, the transcripts of certain gene were reduced during the development of staminate or instaminate organs, such as the genes for protein-unusual-floral organs (Unigene 13103_S7), Apetala2 (Unigene19958_S7), JHL07K02.12 (CL7553.Contig1_S7), RAD54B (CL3359.Contig1_S7), bel1-homeotic-protein (Unigene23982_S7) and Protein-BABY-BOOM (Unigene14170_S7). Other genes exhibited the highest transcript level in the stamen or carpel developing stage of staminate or instaminate organs (stages S2, S4-S5), including Sepallata-1-like-protein (CL3654.Contig1_S7). Accordingly, higher transcripts in both the S1 and S6 stages were observed for more than one-fourth of the transporter DEGs that showed changes in transcript levels during the S1-S6 stages (S4 Fig). Specifically, some genes were up-regulated in the staminate (S1-S2-S3) and instaminate (S1-S4-S5-S6) organ maturation stages, including metal-nicotianamine transporter (transporter of boron, copper, zinc/iron), ATP-binding cassette (transporter of Nramp, hexose, sugar, aromatic and neutral amino acid), cation-transporting ATPase [transporter of aquaporin (PIP and NIP family member)], outward rectifying K+ exchanger, and vacuolar cation/proton exchanger. Members of several large gene families, such as the nitrate transporter family, amino acid family and multidrug resistance protein family, exhibited various changes during floral organogenesis. Transcripts for genes related to phytohormone synthesis or responsive proteins also varied in different floral stages (S5 Fig). Transcripts for the majority of genes related to the synthesis of auxin, ethylene, and jasmonic acid (referred to as lipoxygenase) or response to the stimulation of these three phytohormone were increased at the staminate (S1-S2-S3) and instaminate (S1-S4-S5-S6) organ maturation stages, whereas transcripts of cytochrome (referred to the synthesis of abscisic acid) were reduced at these two stages.

**Fig 5:**
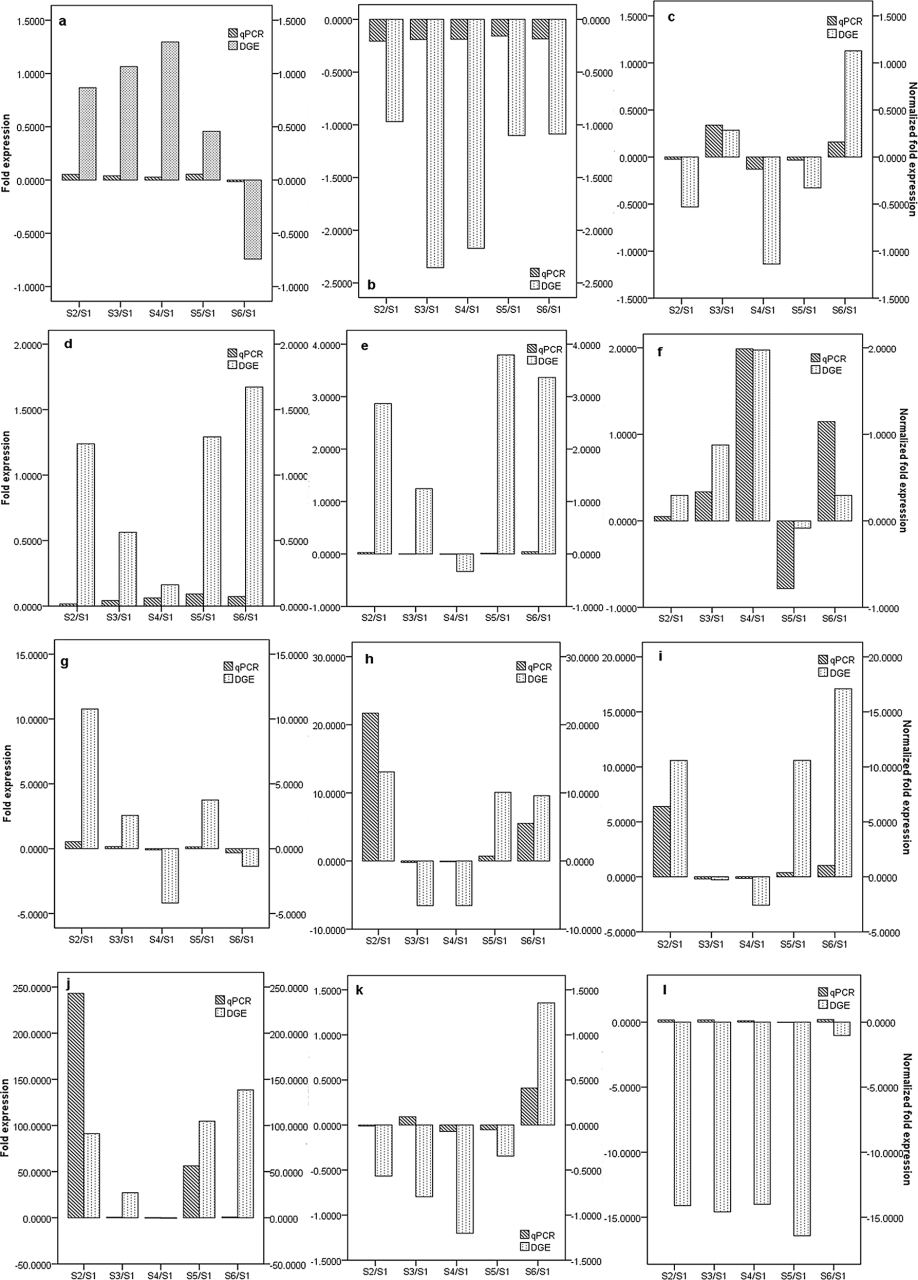
GO classification analysis of the sex-related genes in group 1 (A), group 2 (B) and group 3 (C).

**Fig 6:**
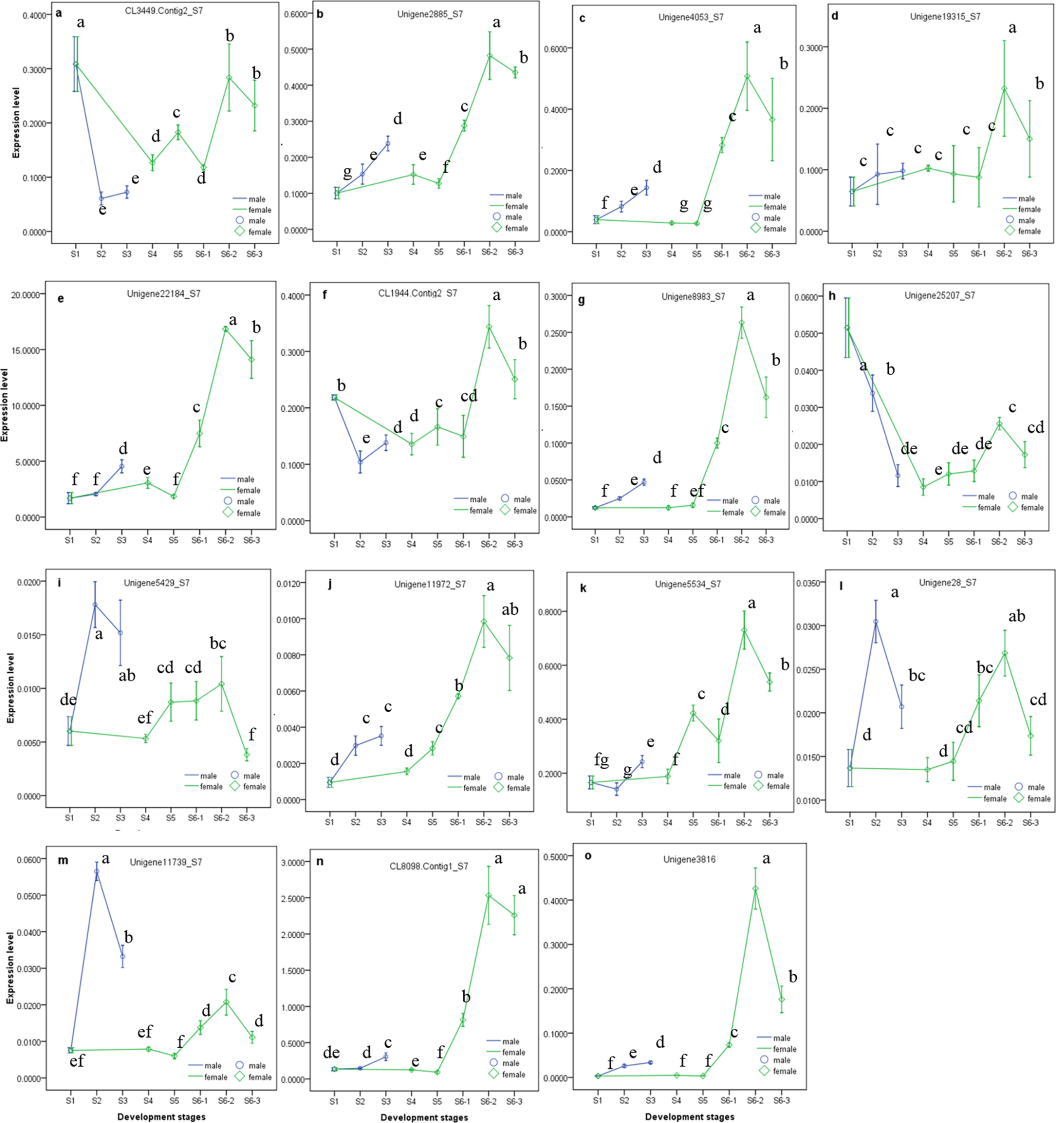
KEGG classification analysis of the sex-related genes in group 1 (A), group 2 (B) and group 3 (C).

Based on flower sex differentiation traits, sex-related genes were screened out and divided into three groups: group 1, genes differently expressed between staminate and instaminate flower at the stamen and carpel differentiation stages (genes showed difference not only in the expression trend between the stages from S1 to S2 in staminate flower and stages from S1 to S4 in instaminate flower but also in the expression level between S2 and S4); group 2, genes differently expressed between staminate and instaminate flower at the stamen and carpel development stage (genes showed difference not only in the expression trend between the stages from S2 to S3 in staminate flower and stages from S4 to S5 in instaminate flower but also in the expression level between S3 and S5); and group 3, genes differently expressed between staminate and instaminate flower at their later development stage (genes showed difference not only in the expression trend between the stages from S2 to S3 in staminate flower and stages from S5 to S6 in instaminate flower but also in the expression level between S3 and S6);

Sex-related genes were screened out from the DEGs, including 85 genes in group 1, 126 in group 2 and 592 in group 3, and then analyzed in the GO and KEGG databases. The molecular functions that only distributed in group 3 included three biological process subcategories (biological adhesion, viral reproduction and pigmentation), three cellular component subcategories (extracellular matrix, extracellular matrix part and membrane-enclosed lumen) and one molecular function subcategory (protein binding transcription factor activity), indicating that these molecular functions are related to the sex differentiation of flowers, such as carpel fusion and stamen degeneration. Locomotion was distributed in groups 1 and 3, suggesting that this process is closely related with stages 2 and 4 of sex differentiation. However, valine, leucine and isoleucine degradation and protein export were only found in group 1, suggesting that these two pathways are involved in carpel and stamen differentiation (Fig 5). Furthermore, being only found in group 3, several pathways (such as brassinosteroid biosynthesis; RNA polymerase; ubiquitin-mediated proteolysis; purine metabolism; pyrimidine metabolism; arginine, proline, cysteine, methionine and tryptophan metabolism; terpenoid biosynthesis; phosphatidylinositol signaling system; and glycan degradation) appear to participate in sex differentiation (such as the development of stamens and flower mature in staminate; carpel fusion and flower mature in instaminate) (Fig 6). On the other hand, the genes showed both different expression levels and opposite expression trends between S2 and S4 or S6 and S3 were screened out as important sex-related genes, obtaining 14 genes (Table 4). The annotation and expression level of these genes were showed in Table 4.

**Table 4:** Annotation of genes with different expression levels and opposite expression trends between male and instaminate flowers in each group. FC, fold change in gene expression level (group 1: log _2_ S4/S2; group 3: log _2_ S6/S3).

gene expression trend	Gene ID	Species	Annotation	Accession No	FC
S6>S5, S6>S3, S2>S3 (group 3)	CL8092.Contig2_S7	*Ricinus communis*	inorganic phosphate transporter, putative	XP_002524622.1	2.503
S6>S5, S6>S3, S2>S3 (group 3)	CL4566.Contig1_S7	* *			5.785
S6>S5, S6>S3, S2>S3 (group 3)	Unigene25538_S7	*Populus trichocarpa*	predicted protein	XP_002329080.1	2.555
S6>S5, S6>S3, S2>S3 (group 3)	Unigene25383_S7	*Ricinus communis*	conserved hypothetical protein	XP_002513940.1	2.631
S6>S5, S6>S3, S2>S3 (group 3)	Unigene8591_S7	*Populus trichocarpa*	predicted protein	XP_002317453.1	3.067
S6>S5, S6>S3, S2>S3 (group 3)	Unigene17053_S7	*Ricinus communis*	protein CRABS CLAW, putative	XP_002512055.1	3.685
S6>S5, S6>S3, S2>S3 (group 3)	Unigene6830_S7	* *			4.672
S6>S5, S6>S3, S2>S3 (group 3)	Unigene17854_S7	*Ricinus communis*	ubiquitin carboxyl-terminal hydrolase, putative	XP_002524120.1	2.118
S6>S5, S6>S3, S2>S3 (group 3)	Unigene16429_S7	*Ricinus communis*	ATP-binding protein, putative	XP_002529046.1	1.329
S5>S6, S3>S6, S3>S2 (group 3)	CL7076.Contig2_S7	*Ricinus communis*	conserved hypothetical protein	XP_002531438.1	-2.55
S5>S6, S3>S6, S3>S2 (group 3)	Unigene917_S7	*Beta vulgaris* subsp. maritima	hypothetical protein BevumaM_p115	YP_004222348.1	-1.585
S5>S6, S3>S6, S3>S2 (group 3)	Unigene22673_S7	*Citrullus lanatus*	cytochrome c oxidase subunit 1	YP_003587228.1	-1.753
S4>S1, S1>S2, S4>S2 (group 1)	CL778.Contig3_S7	*Ricinus communis*	chlorophyll A/B-binding protein, putative	XP_002519724.1	1.037
S4>S1, S1>S2, S4>S2 (group 1)	Unigene4888_S7	*Ricinus communis*	transcription factor, putative	XP_002509493.1	1.572

### Gene expression patterns of selected genes

**Fig 7:**
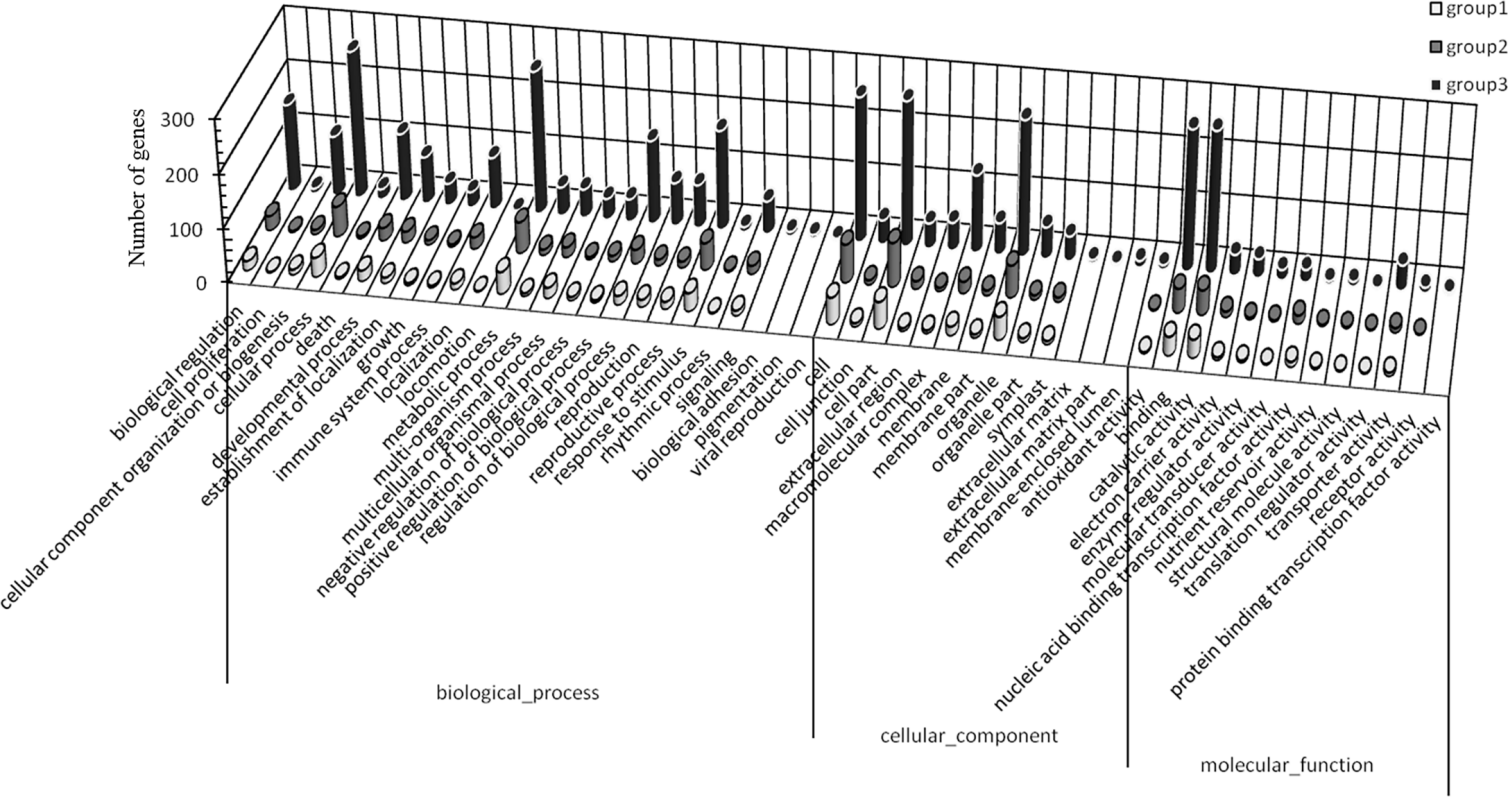
Expression pattern of randomly selected genes. The fold changes of the genes were calculated as the log_2_ value of the S2/S1, S3/S1, S4/S1, S5/S1, and S6/S1 comparisons and are shown on the y-axis (“Fold expression” indicates the fold changes of DGEs, and “Normalized fold expression” indicates the fold changes of the genes by qRT-PCR analysis). (a: CL633.Contig1_S7; b: CL8050.Contig1_S7; c: Unigene24406_S7; d: Unigene17053_S7; e: Unigene8655_S7; f: CL6457.Contig2_S7; g: Unigene26305_S7; h: Unigene25803_S7; i: Unigene169_S7; j: Unigene8845_S7; k: Unigene17081_S7; and l: Unigene17829_S7.).

To validate the expression profiles obtained by RNA-Seq, quantitative real-time PCR (qRT-PCR) was performed on twelve genes selected at random with high or low expression levels. Expression comparisons were performed between S1 and S2, S1 and S3, S1 and S4, S1 and S5, and S1 and S6. For all genes, the trend in qRT-PCR expression agreed with the RNA-Seq data except for that of Unigene17081_S7 and Unigene17829_S7 (Fig 7).

**Fig 8:**
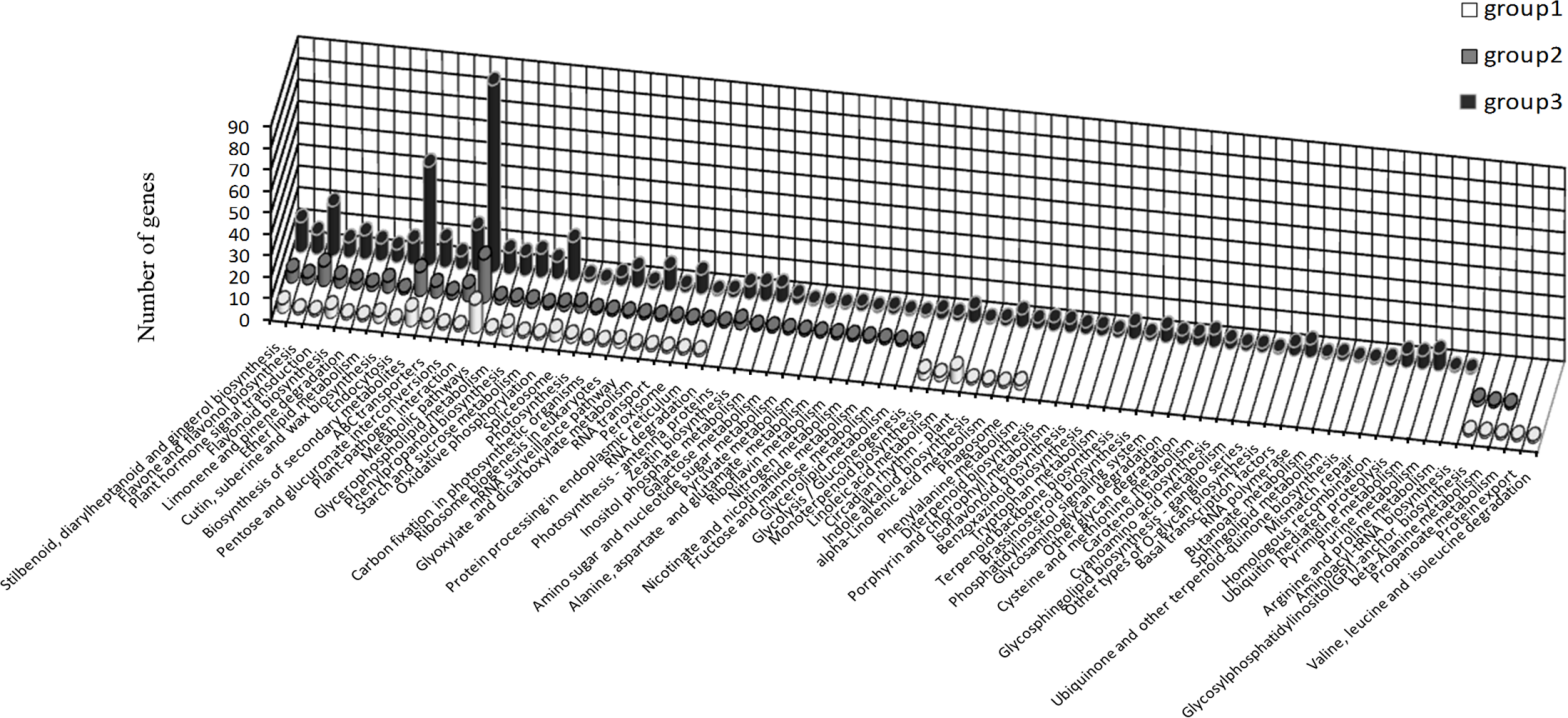
One-way ANOVA analysis expression level of 15 selected genes in samples at different development stages (later stage (S6 stage) of instaminate flower development (or embryo sac development) is divided by S6-1, S6-2 stage, S6-3 stage), as detected by real-time QPCR. Different letters in the same row indicate significant differences (P≤0.005).

The expression patterns of 15 selected genes were shown in Fig 6. Unigene11972 _S7, Unigene3816_S7, Unigene4053_S7, CL8098.Contig1_S7, Unigene22184_S7, Unigene2885_S7 and Unigene8983_S7 exhibited a small degree of up-regulation in S2 and S3, but a large extent of up-regulation was observed at embryo sac development stage of instaminate (S6-1, S6-2 and S6-3; S6 stage for instaminate flower development is divided by S6-1, S6-2 stage, S6-3 stage, as mentioned in Figs 1 and 8). The expressions of Unigene3816_S7 and Unigene8983_S7 changed slightly in S4 and S5, while that of Unigene22184_S7, Unigene2885_S7 and Unigene11972_S7 showed small increases in S4 and/or S5. Unigene4053_S7 and CL8098.Contig1_S7 were slightly down-regulated in S4 and S5, and CL3449.Contig2_S7 and Unigene25207_S7 were greatly down-regulated after S1 in both instaminate and staminate flowers. Unigene5429_S7, Unigene28_S7 and Unigene11739_S7 were markedly up-regulated at S2, followed by a down-regulation at S3 in staminate flowers; but were not significantly changed at S4 and S5, followed by up-regulation at S6-1 and S6-2, and down-regulation at S6-3 in instaminate flowers. CL1944.Contig 2_S7 was down-regulated at S2, and then shown a small up-regulation at S3 in staminate flowers; and firstly down-regulated at S4, and greatly up-regulated at S6-2, followed by a moderate down-regulation at S6-3 in instaminate flowers. The expression of Unigene5534_S7 was not significantly affected in S2 or S4 and showed only a small increase at S3 but a greater increase at S5; after a small decrease at S6-1, this expression greatly increased at S6-2 followed by a small decrease at S6-3. Unigene19315_S7 was only significantly up-regulated at S6-2, followed by a down-regulation at S6-3 in instaminate flowers.

## Discussion

In total, 54,996,594 clean reads were obtained by transcriptome sequence analysis, and 57,962 unigenes were obtained via read assembly. Of these unigenes, 47,423 were successfully annotated (Table 3). To investigate the molecular mechanism of flower sex differentiation, we constructed eighteen DGE libraries from flower buds at different developmental stages to analyze gene expression patterns. The qualities of the DGE libraries were further confirmed by qRT-PCR (Fig 7). In our study, the majority of DEGs for the meristem transition from vegetative to reproductive stage were induced prior to the S1 stage when sex differentiation was not initiated yet, and down-regulated in the reproductive stage (after S2 stage) due to the sensitive cellular regulation system (S3 Fig). A previous study showed that ethylene and auxin were involved in floral organogenesis [12]; similarly, the majority of DEGs related to ethylene and auxin were up-regulated in the male and female organ mature stages (S3 Fig). Certain stress hormone-related genes were also up-regulated in this process, which is consistent with the studies of Liu et al. [13] and Huang et al. [14, 15]. Metal absorption by floral organs would be accelerated during the reproductive stage; thus, boron, copper, and zinc/iron transporters, and a metal-chelating transporter (metal-nicotianamine transporter) were up-regulated (S2 Fig). Based on the DGE analysis, sex-related genes were screened out, including 85 genes in group 1, 126 genes in group 2 and 592 genes in group 3. As sex differentiation progressed, the number of sex-related genes increased (Figs 5 and 6). Several molecular functions and biological pathways were unique to group 3 genes, suggesting that they contribute to the late period of sex differentiation, such as the extracellular matrix, biological adhesion, protein binding transcription factor activity, active base and amino acid metabolism, glycan degradation, protein biosynthesis, brassinosteroid biosynthesis, terpenoid biosynthesis, phosphatidylinositol signaling system and ubiquitin-mediated proteolysis (Figs 5 and 6).

Application of exogenous 6-benzyladenine (6-BA) (a synthetic cytokinin) on inflorescence buds with a diameter about 0.5 cm was reported to increase both the total number of flowers per inflorescence and the instaminate-to-staminate flower ratio in *J*. *curcas* [10]. A further gene expression profiles analysis showed that 6-BA treatment not only up-regulated JcCKX5b (a cytokinin oxidases/dehydrogenases gene), but also down-regulated JcLOG9 (a gene involves in the final step of bioactive cytokinin synthesis), whereas hardly affected other genes that function in cytokinin oxidation or biosynthesis [10], which suggested that exogenous cytokinin application perhaps negatively affected the biosynthesis of endogenous cytokine. Similarly, in present research, a total of five cytokinin oxidases/dehydrogenases genes (Unigene11972_S7, Unigene2716_S7, Unigene13960_S7, Unigene13959_S7, Unigene9593_S7) were identified, and only Unigene11972_S7 was differently expressed between staminate and instaminate flowers, showing a down-regulation at the stages from S2 to S3 in staminate and a up-regulation at the stages from S4 to S6 in instaminate flower. Different from the transcriptome data, the expression level of Unigene11972_S7 determined by qRT-PCR exhibited a significant increase at stamen development stage in staminate flower, and a sharper and larger increase after carpel differentiation stage in instaminate flower (Fig 8J). Pan et al (2014) and our study suggested that endogenous cytokinin would be decreased during sex differentiation and development of *J*. *curcas*. To elucidate the role of dogenous cytokinin during sex differentiation and development of *J*. *curcas*, it is necessary to study the endogenous cytokinin level and the expression pattern of the genes involved in cytokinin biosynthesis, metabolism and signaling of flower bud at different stage of sex differentiation.

Gibberellic acid (GA) was expected to play important roles in flower sex differentiation in *J*. *curcas*. AGAMOUS-like 20 (AGL20), a gene that is downstream of *FLC*, is either positively regulated by *CO* and GA or negatively regulated by *FLC* [16], and acts as an important integrator of four pathways (vernalization, autonomous, photoperiod and GA-dependent pathways) controlling flowering in *Arabidopsis*. GID1 positively regulates the GA-signaling cascade in *Arabidopsis*. GID1 forms a complex with GA (GID1-GA) and then directly interacts with DELLA protein (a repressor of GA signaling), promoting interaction between DELLA and SLY1 (a component of the SCF^SLY1^ E3 ubiquitin ligase) and triggering the degradation of DELLA [17, 18]. In addition, GASA4 can be up-regulated by GA in meristematic regions and is involved in floral meristem identification, early flower development, and style and stamen filament maturation [19, 20]. In *J*. *curcas*, AGL20, GID1 and GASA4 perhaps participate in the embryo sac development of instaminate flowers (the stages from carpel begain to fuse to flower mature, such as S6-1, S6-2 and S6-3), while GASA4 also functions in stamen differentiation. AGL20 (CL3449.Contig2_S7) exhibited a marked down-regulation after S1, a slight up-regulation trend at S5, and a notable up-regulation at S6-2 (Fig 8A). GID1 (Unigene5534_S7) was greatly up-regulated after the three carpels became distinct in instaminate flowers (S4) (Fig 8K); and GASA4 (Unigene5429_S7) was up-regulated at S2 and S6-2 stage (Fig 8I). Furthermore, a transcription factor (Unigene28_S7) that negatively regulates the GA signaling pathway was up-regulated at the stage of stamen differentiation (S2) (Fig 8L). In addition, the RING-H2 finger protein ATL3J (CL8098.Contig1_S7) and r2r3-myb transcription factor (Unigene3816_S7) were expected to function in the embryo sac development stage of instaminate flowers in *J*. *curcas*, and both genes were found to indeed be up-regulated at these stages (S6-1, S6-2 and S6-3) (Fig 8N and 8O). However, the slight up-regulation of r2r3-myb at S2 and S3 suggested that it also functioned in the differentiation and development of stamens (Fig 8O). RING finger protein, a subfamily of ubiquitin protein ligase E3, plays key roles in regulating growth/developmental processes and might be directly involved in the regulation of hormone signaling pathways, including that of GA [21–24]. R2R3-MYB genes, members of a large family, are involved in the signal transduction pathways of salicylic acid [25], abscisic acid [26], GA [27, 28], and jasmonic acid [16], and play roles in sperm cell specification [29], stamen and pollen maturation [30], and anther tapetum and stigma papillae development.

Primary/early auxin response genes consist of members of three gene families, AUX/IAA, SAUR (small auxin up RNA) and GH3 (gretchen hagen3) [31]; the first two may participate in flower sex differentiation in *J*. *curcas*. The auxin-signaling pathway as mediated by Aux/IAA1 and ARF is involved in *J*. *curcas* at its embryo sac development (S6 stage). Auxin-induced protein 22D (Unigene25207_S7) (belonging to the Aux/IAA1 family) and ARF6-like protein (CL1944.Contig2_S7) were firstly down-regulated after S1 and then up-regulated at S6-2 (Fig 8H and 8F). Aux/IAA proteins have been suggested to repress auxin-inducible transcription by negatively regulating the transcriptional activity of auxin response factor (ARF) [32], and could be removed by an auxin stimulus through the proteolysis of Aux/IAAs, triggered by the SCF^TIR1/AFB^ ubiquitin ligase. ARF6 also regulates multiple events together with ARF8, including inflorescence stem elongation, stamen filament elongation, anther dehiscence, stigmatic papillae elongation, gynoecium maturation and flower bud opening [33]. Furthermore, ARF6 transcripts are cleavage targets of the microRNA *miR167* which can be induced by exogenous auxin [34, 35], and the post-transcriptional cleavage of ARF mRNAs by microRNA also plays an important role in auxin signaling [36–38]. Therefore, the function of this ARF6-like protein in sex differentiation requires further study. In addition, SAUR which could be induced by auxin treatment [39, 40] is correlated with auxin-mediated cell elongation [41–45] and negatively influences auxin biosynthesis and transport in rice [46]. In *J*. *curcas*, SAUR may promote the differentiation and maturation of stamens and embryo sac. Auxin-induced protein X10A (Unigene8983_S7) (a member of the SAUR family) was up-regulated at S2, S3, S6-1, S6-2 and S6-3 (Fig 8G). ARP1-like protein (Unigene22184_S7) may regulate pollen maturation in staminate flowers (S3), carpel differentiation (S4), and stigma maturation in instaminate flowers (S6-2 and S6-3), as suggested by its up-regulation at these stages (Fig 8E). ARP1 can be repressed or induced by auxin [47–49] and is highly expressed during pollen maturation in tobacco [50].

Pentatricopeptide repeat-containing (PPR) proteins mediate specific RNA-processing events, including RNA editing [51], transcript processing [52], and translation initiation [53], and are involved in the restoration of cytoplasmic male sterility in the mitochondria [54–56] as well as the first mitotic division during male or female gametogenesis [57]. A PPR protein (Unigene2885_S7) might be involved in the differentiation of the stamen and carpel, and embryo sac development at its later stage, as suggested by a small up-regulation at stages S2 and S4 and a large up-regulation at embryo sac development stage of instaminate flower (S6-1, S6-2 and S6-3) (Fig 8B). CLAVATA1 (*CLV*1) can restrict *WUSCHEL* gene expression to maintain shoot and floral meristems [58–62] and control the number of carpels in the floral meristem [63]. In *J*. *curcas*, two homologous *CLV*1 genes (Unigene19315_S7 and Unigene4053_S7) appear to be involved in the embryo sac development (S6-2, S6-3 and/or S6-1). However, the up-regulation of Unigene4053_S7 in S2 and S3 suggested that this gene perhaps also functioned in stamen development (Fig 8C and 8D). On the other hand, an AMP-activated protein kinase (Unigene11739_S7) is expected to involve in the development of staminate flowers (S2, 3) and the embryo sac of instaminate flower (S6-1, S6-2 and S6-3) (Fig 8M), as suggested by its up-regulation at these stages.

The genes with different expression levels and opposite expression trends between S2 and S4 or S6 and S3 were screened out as important sex-related genes (Table 4). Of the 14 genes obtained, 2 genes (CL4566.Contig1_S7 and Unigene6830_S7) could not be annotated, and 7 genes had functional annotation based on sequence similarity, including CL8092.Contig2_S7 (inorganic phosphate transporter), unigene17854_S7 (ubiquitin carboxyl-terminal hydrolase), Unigene17053_S7 (CRABS CLAW), Unigene16429_S7 (ATP-binding protein), Unigene22673_S7 (cytochrome C oxidase subunit 1), CL778.Contig3_S7 (chlorophyll A/B-binding protein) and Unigene4888 (transcription factor). Of these seven genes, only CRABS CLAW has been reported to function in the sex differentiation of flowers, such as in carpel morphogenesis [64, 65] and in the development of ovules and stamens, and is expected to have similar functions in *J*. *curcas* sex differentiation. Of the remaining six genes, chlorophyll A/B-binding protein and Unigene4888_S7 contribute to carpel differentiation; ubiquitin carboxyl-terminal hydrolase and inorganic phosphate transporter contribute to the embryo sac development; Unigene16429_S7 is expected to promote stamen degeneration at later development stages of instaminate flower; and cytochrome C oxidase subunit 1 contributes to the development of the stamen.

## Conclusions

A total of 57,962 assembled unigenes were obtained using Illumina sequencing technology, and 47,423 unigenes were annotated. DGE library analysis provided comprehensive, valuable information regarding the sex differentiation of flowers in *J*. *curcas*. The expression pattern analysis using real-time qPCR suggested the following: GASA4, *CLV*1 (Unigene4053_S7) and AMP-activated protein kinase contribute to stamen differentiation; ARF6-like protein, AGL20, RING-H2 finger protein ATL3J, auxin-induced protein 22D, *CLV*1 (Unigene19315_S7) and r2r3-myb transcription factor contribute to development of mbryo sac in instaminate flower; and cytokinin oxidase, a transcription factor, ARP1-like protein, GID1 and X10A, function in both stamen differentiation and embryo sac development. GA signaling pathways contribute not only to the differentiation and development of stamens, but also to the development of embryo sac. In addition, auxin signaling pathways were thought to involve in stamen development, and also were important for the embryo sac development. Overall, our data provide valuable new clues and information about the sex differentiation of flowers in *J*. *curcas*, and further study on the functions of sex-related genes will be helpful in eluciding the sex differentiation mechanism of *J*. *curcas*.

## Material and Methods

### Flower sample collection

Mixed flower buds or inflorescences at different developmental stages of *J*. *curcas* were collected from thirty trees in Zhenfeng, Guizhou Province, China (36°14′50.2″N, 87°51′47.8″E) (*J*. *curcas* is not an endangered or protected species, and no specific permission is required for its collection.). The flower buds used for microscopic observation were fixed immediately in formaldehyde-acetic acid-50% alcohol mixtures (4: 6: 90, v/v), and those used for transcriptome analysis were dipped immediately in RNAlocker (Tiandz, Inc, Beijing China) on ice.

### Observation of flower buds at different developmental stages

For microscopic observation, after having been fixed in formaldehyde-acetic acid-50% alcohol mixtures for 27 h, the buds or inflorescences then were transferred to 70% ethanol for storage; thereafter, buds were anatomized in 95% ethanol under an optical anatomical lens. For SEM (scanning electron microscope) study, the buds selected under optical anatomical lens were desiccated through a graded series of alcohol–isoamyl acetate, coated with palladium using a Hitachi E-1010 at 15 mA. Flower organs were then observed with a Hitachi S-4800 SEM at 10.0 kV.

### Flower sample collection and total RNA extraction

The flower buds stored in RNAlocker were then sorted according to development phases (stage 1: sex differentiation is not initiated; stage 2: from stamen primordia beginning to differentiate to ten stamen primordia formed; stage 3: from ten stamen primordia formed to mature staminate flowers; stage 4: from carpel primordia beginning to differentiate to three distinct carpels formed; stage 5: carpels continue developing; stage 6: from carpels beginning to fuse to mature instaminate flowers.) using an optical anatomical lens (Fig 1). For total RNA extraction, flower buds in the six different developmental phases were ground in liquid nitrogen, and total RNA was isolated using E.A.N.A Plant RNA Kit (Omega Bio-Tek, Inc, Norcross GA USA) according to the manufacturer’s manual.

### Library preparation of developing flower for transcriptome analysis

RNA samples (named S7) from flower buds in six different developmental phases (as described above) were pooled for transcriptome analysis to obtain comprehensive gene expression information during the process of flower sex differentiation. After DNase I treatment, magnetic beads with oligo (dT) were used to isolate mRNA. The mRNA was then fragmented into short fragments in fragmentation buffer, and cDNA was synthesized using the mRNA fragments as templates. The resulting short cDNA fragments were purified with a QIAquick PCR extraction kit and resolved in EB buffer. After the fragment ends were repaired and poly (A) tailed, the short fragments were ligated to sequencing adapters. Suitable fragments were selected as templates for PCR amplification. During the QC steps, an Agilent 2100 Bioanalyzer and an ABI Step OnePlus Real-Time PCR System were used for the quantification and qualification of the sample library. Sequencing of the library was performed using an Illumina HiSeq™ 2000 at Beijing Genome Institute (Shenzhen, China).

### Illumina sequencing, *de novo* assembly of reads, and unigene annotation

The raw reads produced from the sequencing machines were filtered by removing adaptor sequences, low-quality reads, and reads with a percentage of unknown nucleotides of more than 5%. The *de novo* assembly of clean reads into unigene was carried out using a short reads assembling program: Trinity [66]. The resulting sequences were called unigenes. Gene family clustering then was performed, and the unigenes were divided into two classes. One class included clusters with the prefix CL followed by the cluster id. There were several unigenes in one cluster, and the similarity between them was more than 70%. The other class included singletons with the prefix unigene. A Blastx alignment (E-value < 1.0E^-5^) was carried out between the unigenes and protein databases (NR, Swiss-Prot, KEGG and COG), and the best alignment results were used for the sequence direction determination of the unigenes. When the results of different databases conflicted with each other, a priority order of NR, Swiss-Prot, KEGG and COG was followed to decide the sequence direction of the unigenes. When a unigene was not aligned with any of the above databases, its sequence direction was determined using ESTScan software.

### DGE library preparation and sequencing

Total RNA from flower bud samples in six different development stages (S1, S2, S3, S4, S5, and S6) was first treated with DNase I to degrade any possible contaminating DNA, and then the mRNA was enriched using oligo (dT) magnetic beads. The obtained mRNA was fragmented into short fragments (approximately 200 bp) in fragmentation buffer. First-strand cDNA was synthesized using random-hexamer primers, and the second strand was synthesized using the first strand as template. The double-stranded cDNA was purified with magnetic beads. End reparation and 3’-end single nucleotide A (adenine) addition were then performed. Finally, sequencing adaptors were ligated to the fragments, and the fragments were enriched by PCR amplification. During the QC step, an Agilent 2100 Bioanalyzer and an ABI StepOnePlus Real-Time PCR System were used to qualify and quantify the sample library. The library products were then ready for sequencing via Illumina HiSeq^TM^ 2000.

### DGE analysis

By base calling, the original image data produced by sequencing were transferred into sequences (raw reads). To obtain clean reads for further analysis, the raw reads were filtered by removing adaptor sequences, low-quality reads, and reads with a percentage of unknown bases (N) of more than 10%. The clean reads were then mapped to the transcriptome of a developing flower reference database using SOAPaligner/SOAP2, and no more than 2 mismatches were allowed in the alignment. The expression level of each gene was determined by the numbers of reads that were uniquely mapped to the specific gene and the total number of uniquely mapped reads in the sample. The gene expression level was calculated using RPKM method (reads per kb per million reads) [67]. If there was more than one transcript for a gene, the longest transcript was used to calculate its expression level and coverage.

A false discovery rate (FDR) ≤ 0.001 and an absolute value of log_2_Ratio ≥ 1 were used as the threshold to determine the DEGs. The DGEs were then subjected to GO and KEGG Ontology (KO) enrichment analysis using hypergeometric testing. The enriched P-values were calculated as follows: $\text{P}=1\text{-}\sum_{\text{i}=0}^{\text{m-}1}\frac{\left(\begin{array}{l} \text{M}\\ \text{i} \end{array}\right)\left(\begin{array}{l} \text{N-M}\\ \text{n-i} \end{array}\right)}{\left(\begin{array}{l} \text{N}\\ \text{n} \end{array}\right)}$, where N is the number of genes with GO or KO annotation; n is the number of DEGs in N; M is the number of genes in certain GO or KO terms; and m is the number of DEGs in M. A Bonferroni correction was imposed on the P-value, taking the corrected P-value ≤ 0.05 as a threshold.

### Quantitative real-time PCR (qRT-PCR) analysis

To confirm the validation of DGEs, total RNA was isolated from the same samples used for DGE establishment. Moreover, total RNA was extracted from flower buds at eight different development phases (S1, S2, S3, S4, S5, S6 stage, and S6 stage are further divided into S6-1, S6-2 and S6-3 stage), and used for expression pattern analysis of 15 selected sex-related genes (Fig 1). The total RNA (1 μg) of each sample was used for first-strand cDNA synthesis using AMV RNA PCR Kit 3.0 (Takara). These cDNA samples were then used for real-time PCR using an ABI StepOnePlus Real-Time PCR System (Applied Biosystems, Inc. USA) with 2× SYBR green PCR mix (QIAGEN, Shanghai, China). The cycling procedure was 95°C for 3 min, followed by 40 cycles of 94°C for 10 s, 59°C for 10 s and 72°C for 40 s. The β-tubulin gene was chosen as the endogenous reference gene for the qPCR analysis. The sequences of the applied primers are given in supporting information S1 File (Tables A and B). Three replicates were analyzed for each gene. The average threshold cycle (Ct) was calculated, and the relative expression level of each gene was then calculated according to the 2^-⊿⊿Ct^ method. A one-way ANOVA analysis of the gene expression level of the samples at different development stages was performed using the software Statistical Package for the Social Science (SPSS) version 11.5 (SPSS Inc., Chicago, IL, USA). The data were subjected to log-transformation if necessary. The individual treatment means were compared using the LSD (least significance difference) test.

## Supporting Information

S1 File Primers for the genes and reference gene used for the validation of expression profiles (Table A). Primers for the genes and reference gene used for the expression pattern analysis (Table B).
(DOC)

S1 Fig The sequencing saturation and distribution of the transcript with the distance from 5`ends for the eighteen samples. (TIF)

S2 Fig Correlation between the biological replicates of same development stage for the floral samples in different development stages. (TIF)

S3 Fig Cluster analysis showing the differentially expressed genes involved in floral development and differentiation from stages S1 to S6. (TIF)

S4 Fig Cluster analysis showing the differentially expressed genes involved in the transport of cellular ions and compounds from stages S1 to S6. (TIF)

S5 Fig Cluster analysis showing the differentially expressed genes involved in phytohormone synthesis and metabolism from stages S1 to S6. (TIF)

## References

[1] Heller J (1996) Physic nut. *Jatropha curcas* L. Promoting the conservation and use of underutilized and neglected crops. 1.PhD dissertation, Institute of Plant Genetic and Crop Plant Research, Gatersleben, Germany, and International Plant Genetic Resource Institute, Rome, Italy.

[2] Francis G, Edinger R, Becker K (2005) A concept for simultaneous wasteland reclamation, fuel production, and socio-economic development in degraded areas in India: need, potential and perspectives of Jatropha plantations. Nat Resour Forum 29: 12–24.

[3] Tewari DN (2007) Jatropha and Biodiesel, 1st edn. Ocean Books Ltd, New Delhi.

[4] Foidl N, Foidl G, Sanchez M, Mittelbach M, Hackel S (1996) *Jatropha curcas* L. as a source for the production of biofuel in Nicaragua. Bioresour Technol 58: 77–82.

[5] Dehgan B, Webster G (1992) Morphology and in frageneric relationships of the genus J. curcas University of California Press, Berkeley, CA, USA.

[6] Carvalhoa CR, Clarindo WR, Praca MM, Araújo FS, Carels N (2008) Genome size, base composition and karyotype of *Jatropha curcas* L., an important biofuel plant. Plant Sci 174: 613–617.

[7] Natarajan P, Kanagasabapathy D, Gunadayalan G, Panchalingam J, Shree N, Sugantham PA, et al (2010) Gene discovery from Jatropha curcas by sequencing of ESTs from normalized and full-length enriched cDNA library from developing seeds. BMC Genomics, 11:606 10.1186/1471-2164-11-606 20979643 PMC3091748

[8] Costa GG, Cardoso KC, Del Bem LE, Lima AC, Cunha MAS, Campos-Leite L, et al (2010) Transcriptome analysis of the oil-rich seed of the bioenergy crop *Jatropha curcas* L. BMC Genomics 11: 462–471. 10.1186/1471-2164-11-462 20691070 PMC3091658

[9] Wang W, Wei B, Pun S, Qin DJ, Wang WS, Zhang SH, et al (2011) Profilling of gene expression in the reproductive organs of *Jatropha curcas*. China Biotechnol 31: 38–48.

[10] Pan BZ, Chen MS, Ni J and Xu ZF (2014) Transcriptome of the inflorescence meristems of the biofuel plant Jatropha curcas treated with cytokinin. BMC Genomics 15: 974–993 10.1186/1471-2164-15-974 25400171 PMC4246439

[11] Sato S, Hirakawa H, Isobe S, Fukai E, Watanabe A, Kato M, et al (2011) Sequence analysis of the genome of an oil-bearing tree, *Jatropha curcas* L. DNA Res 18: 65–76. 10.1093/dnares/dsq030 21149391 PMC3041505

[12] Ruonala R, Rinne PLH, Baghour M, Moritz T, Tuominen H and Kangasjärvi J (2006) Transitions in the functioning of the shoot apical meristem in birch (Betula pendula) involve ethylene. Plant J 46: 628–640. 16640599 10.1111/j.1365-313X.2006.02722.x

[13] Liu LYD, Tseng HI, Lin CP, Lin YY, Huang YH, Huang CK, et al (2014) High-throughput transcriptome analysis of the leafy flower transition of *Catharanthus roseus* induced by peanut witches'-broom phytoplasma infection. Plant Cell Physiol 55: 942–957. 10.1093/pcp/pcu029 24492256

[14] Huang YJ, Liu LL, Huang JQ, Wang ZJ, Chen FF, Zhang QX, et al (2013) Use of transcriptome sequencing to understand the pistillate flowering in hickory (*Carya cathayensis* Sarg.). BMC Genomics 14: 691 10.1186/1471-2164-14-691 24106755 PMC3853572

[15] Huang S, Cerny RE, Qi Y, Bhat D, Aydt CM, Hanson DD, et al (2003) Transgenic studies on the involvement of cytokinin and gibberellin in male development. Plant Physiol 131: 1270–1282. 12644677 10.1104/pp.102.018598 PMC166887

[16] Lee MW, Qi M, Yang Y (2001) A novel jasmonic acid-inducible rice myb gene associates with fungal infection and host cell death. Mol Plant Microbe Interact 14: 527–535. 11310740 10.1094/MPMI.2001.14.4.527

[17] Griffiths J, Murase K, Rieu I, Zentella R, Zhang Z, Powers SJ, et al (2006) Genetic characterization and functional analysis of the gid1 gibberellin receptors in *Arabidopsis*. Plant Cell 18: 3399–3414. 17194763 10.1105/tpc.106.047415 PMC1785415

[18] Ueguchi-Tanaka M, Nakajima M, Katoh E, Ohmiya H, Asano K, Saji S, et al (2007) Molecular interactions of a soluble gibberellin receptor, GID1, with a Rice DELLA protein, SLR1, and gibberellins. Plant Cell 19: 2140–2155. 17644730 10.1105/tpc.106.043729 PMC1955699

[19] Aubert D, Chevillard M, Dorne AM, Arlaud G, Herzog M (1998) Expression patterns of GASA genes in *Arabidopsis thaliana*: the GASA4 gene is up-regulated by gibberellins in meristematic regions. Plant Mol Biol 36: 871–883. 9520278 10.1023/a:1005938624418

[20] Roxrud I, Lid SE, Fletcher JC, Schmidt ED, Opsahl-Sorteberg HG (2007) GASA4, one of the 14-member *Arabidopsis* GASA family of smallpolypeptides, regulates flowering and seed development. Plant Cell Physiol 48: 471–483. 17284469 10.1093/pcp/pcm016

[21] Liu H, Zhang H, Yang Y, Li G, Yang Y, Wang X, et al (2008) Functional analysis reveals pleiotropic effects of rice RING-H2 finger protein gene OsBIRF1 on regulation of growth and defense responses against abiotic and biotic stresses. Plant Mol Biol 68: 17–30. 10.1007/s11103-008-9349-x 18496756

[22] Xie Q, Guo HS, Dallman G, Fang SY, Weissman AM, Chua NH (2002) SINAT5 promotes ubiquitin-related degradation of NAC1 to attenuate auxin signals. Nature 419: 167–170. 12226665 10.1038/nature00998

[23] Zhang X, Garreton V, Chua NH (2005) The AIP2 E3 ligase acts as a novel negative regulator of ABA signaling by promoting ABI3 degradation. Genes Dev 19: 1532–1543. 15998807 10.1101/gad.1318705 PMC1172060

[24] Molnar G, Bancos S, Nagy F, Szekeres M (2002) Characterisation of BRH1, a brassinosteroid-responsive RING-H2 gene from *Arabidopsis thaliana*. Planta 215: 127–133. 12012249 10.1007/s00425-001-0723-z

[25] Raffaele S, Rivas S, Roby D (2006) An essential role for salicylic acid in AtMYB30-mediated control of the hypersensitive cell death program in *Arabidopsis*. FEBS Lett 580: 3498–3504. 16730712 10.1016/j.febslet.2006.05.027

[26] Abe H, Urao T, Ito T, Seki M, Shinozaki K, Yamaguchi-Shinozaki K (2003) *Arabidopsis* AtMYC2 (bHLH) and AtMYB2 (MYB) function as transcriptional activators in abscisic acid signaling. Plant Cell 15: 63–78. 12509522 10.1105/tpc.006130 PMC143451

[27] Murray F, Kalla R, Jacobsen J, Gubler F (2003) A role for HvGAMYB in anther development. Plant J 33: 481–491. 12581306 10.1046/j.1365-313x.2003.01641.x

[28] Gocal GF, Poole AT, Gubler F, Watts RJ, Blundell C, King RW (1999) Long-day up-regulation of a *GAMYB* gene during *Lolium temulentum*in florescence formation. Plant Physiol 119: 1271–1278. 10198085 10.1104/pp.119.4.1271 PMC32011

[29] Borg M, Brownfield L, Khatab H, Sidorova A, Lingaya M, Twell D (2011) The R2R3 MYB transcription factor DUO1 activates a male germline-specific regulon essential for sperm cell differentiation in *Arabidopsis*. Plant Cell 23: 534–549. 10.1105/tpc.110.081059 21285328 PMC3077786

[30] Mandaokar A, Browse J (2009) MYB108 Acts Together with MYB24 to Regulate Jasmonate-Mediated Stamen Maturationin *Arabidopsis*. Plant Physiol 149: 851–862. 10.1104/pp.108.132597 19091873 PMC2633834

[31] Hagen G, Guilfoyle T (2002) Auxin-responsive gene expression: Genes, promoters and regulatory factors. Plant Mol Biol 49: 373–385. 12036261

[32] Ulmasov T, Murfett J, Hagen G, Guilfoyle TJ (1997) Aux/IAA proteins repress expression of reporter genes containing natural and highly active synthetic auxin response elements. Plant Cell 9: 1963–1971. 9401121 10.1105/tpc.9.11.1963 PMC157050

[33] Nagpal P, Ellis CM, Weber H, Ploense SE, Barkawi LS, Guilfoyle TJ, et al (2005) Auxin response factors ARF6 and ARF8 promote jasmonic acid production and flower maturation. Development 132: 107–118. 10.1242/dev.01955 16107481

[34] Kasschau KD, Xie Z, Allen E, Llave C, Chapman EJ, Krizan KA, et al (2003) P1/HC-Pro, a viral suppressor of RNA-silencing, interferes with *Arabidopsis* development and miRNA function. Dev Cell 4: 205–217. 12586064 10.1016/s1534-5807(03)00025-x

[35] Mallory AC, Bartel DP, Bartel B (2005) MicroRNA-directed regulation of Arabidopsis AUXIN RESPONSE FACTOR17 is essential for proper development and modulates expression of early auxin response genes. Plant Cell 17: 1360–1375. 15829600 10.1105/tpc.105.031716 PMC1091760

[36] Wang Z, Liang Y, Li C, Xu Y, Lan L, Zhao D, et al (2005) Microarray analysis for gene expression involved in anther development in rice (*Oryza sativa* L.). Plant Mol Biol 58: 721–737. 16158245 10.1007/s11103-005-8267-4

[37] Wu M, Tian Q, Reed JW (2006) *Arabidopsis microRNA167* controls patterns of *ARF6* and *ARF8* expression, and regulates both female and male reproduction. Development 133: 4211–4218. 17021043 10.1242/dev.02602

[38] Yang J, So Han J, Yoon EK, Lee WS (2006) Evidence of an auxin signal pathway, microRNA167-ARF8-GH3, and its response to exogenous auxin in cultured rice cells. Nucleic Acids Res 34: 1892–1899. 16598073 10.1093/nar/gkl118 PMC1447648

[39] McClure BA, Guilfoyle TJ (1987) Characterization of a class of small auxin-inducible soybean polyadenylated RNAs. Plant Mol Biol 9: 611–623. 10.1007/BF00020537 24277197

[40] Kong Y, Zhu Y, Gao C, She W, Lin W, Chen Y, et al (2013) Tissue-specific expression of *SMALL AUXIN UP RNA41* differentially regulates cell expansion and root meristem patterning in *Arabidopsis*. Plant Cell Physiol 54: 609–621. 10.1093/pcp/pct028 23396598

[41] Spartz AK, Lee SH, Wenger JP, Gonzalez N, Itoh H, Inzé D, et al (2012) The SAUR19 subfamily of SMALL AUXIN UP RNA genes promotes cell expansion. Plant J 70: 978–990. 10.1111/j.1365-313X.2012.04946.x 22348445 PMC3481998

[42] Chae K, Isaacs CG, Reeves PH, Maloney GS, Muday GK, Nagpal P, et al (2012) Arabidopsis SMALL AUXIN UP RNA63 promotes hypocotyl and stamen filament elongation. Plant J 71: 684–697. 10.1111/j.1365-313X.2012.05024.x 22507274

[43] McClure BA, Guilfoyle TJ (1989) Rapid redistribution of auxin-regulated RNAs during gravitropism. Science 243: 91–93. 11540631 10.1126/science.11540631

[44] Gee MA, Hagen G, Guilfoyle TJ (1991) Tissue-specific and organ-specific expression of soybean auxin-responsive transcripts GH3 and SAURs. Plant Cell 3: 419–430. 1840920 10.1105/tpc.3.4.419 PMC160011

[45] Kant S, Bi YM, Zhu T, Rothstein SJ (2009) SAUR39, a small auxin-up RNA gene, acts as a negative regulator of auxin synthesis and transport in rice. Plant Physiol 151: 691–701. 10.1104/pp.109.143875 19700562 PMC2754634

[46] Kant S, Rothstein S (2009) Auxin-responsive SAUR39 gene modulates auxin level in rice. Plant Signal Behav 4: 68–70. 10.4161/psb.4.12.10043 PMC2819449 20514239

[47] Park S, Han KH (2003) An auxin-repressed gene (RpARP) from black locust (*Robinia pseudoacacia*) is posttranscription ally regulated and negatively associated with shoot elongation. Tree Physiol 23: 815–823. 12865247 10.1093/treephys/23.12.815

[48] Reddy ASN, Poovaiah BW (1990) Molecular cloningand sequencing of a cDNA for an auxin-repressed mRNA: correlation between fruit growth and repression of the auxin-regulated gene. Plant Mol Biol 14: 127–136. 2101687 10.1007/BF00018554

[49] Kim HB, Lee H, Oh CJ, Lee NH, An CS (2007) Expression of EuNOD-ARP1 encoding auxin-repressed protein homolog is up-regulated by auxin and localized to the fixation zone in root nodules of *Elaeagnus umbellate*. Mol Cells 23: 115–121. 17464220

[50] Steiner C, Bauer J, Amrhein N, Bucher M (2003) Two novelgenes are differentially expressed during early germination of the male gametophyte of *Nicotiana tabacum*, Biochim Biophys Acta 1625: 123–133. 12531471 10.1016/s0167-4781(02)00598-5

[51] Kotera E, Tasaka M, Shikanai T (2005) A pentatricopeptide repeat proteinis essential for RNA editing in chloroplasts. Nature 433: 326–330. 15662426 10.1038/nature03229

[52] Nakamura T, Schuster G, Sugiura M, Sugita M (2004) Chloroplast RNA binding and pentatricopeptide repeat proteins. Biochem Soc Trans 32: 571–574. 15270678 10.1042/BST0320571

[53] Schmitz-Linneweber C, Williams-Carrier R, Barkan A (2005) RNA immunoprecipitation and microarray analysis show a chloroplast pentatricopeptide repeat protein to be associated with the 5' region of mRNAs whose translation it activates. Plant Cell 17: 2791–2804. 16141451 10.1105/tpc.105.034454 PMC1242273

[54] Bentolila S, Alfonso A, Hanson M (2002) A pentatricopeptide repeat containinggene restores fertility to cytoplasmic male-sterile plants. Proc Natl Acad Sci USA 99: 10887–10892. 12136123 10.1073/pnas.102301599 PMC125068

[55] Kazama T, Toriyama K (2003) A pentatricopeptide repeat-containing gene that promotes the processing of aberrant *atp6* RNA of cytoplasmic male-sterile rice. FEBS Lett 544: 99–102. 12782297 10.1016/s0014-5793(03)00480-0

[56] Brown G, Formanova N, Jin H, Wargachuk R, Dendy C, Patil P, et al (2003) The radish *Rfo* restorer gene of Ogura cytoplasmic male sterility encodes a protein with multiple pentatricopeptide repeats. Plant J 35: 262–272. 12848830 10.1046/j.1365-313x.2003.01799.x

[57] Lu Y, Li C, Wang H, Chen H, Berg H, Xia Y (2011) AtPPR2, an *Arabidopsis* pentatricopeptide repeat protein, binds to plastid 23S rRNA and plays an important role in the first mitotic division during gametogenesis andin cell proliferation during embryogenesis. Plant J 67: 13–25. 10.1111/j.1365-313X.2011.04569.x 21435048 PMC3214271

[58] Clark SE, Running MP, Meyerowitz E (1993) *CLAVATA1*, a regulator of meristem and flower development in *Arabidopsis*. Development 119: 397–418. 8287795 10.1242/dev.119.2.397

[59] Clark SE, Williams RW, Meyerowitz EM (1997) The *CLAVATA1* gene encodes a putative receptor kinase that controls shoot and floral meristem size in *Arabidopsis*. Cell 89: 575–585. 9160749 10.1016/s0092-8674(00)80239-1

[60] Kayes JM, Clark SE (1998) *CLAVATA2*, a regulator of meristem and organ development in *Arabidopsis*. Development 125: 3843–3851. 9729492 10.1242/dev.125.19.3843

[61] Jeong S, Trotochaud AE, Clark SE (1999) The *Arabidopsis CLAVATA2* gene encodes a receptor-like protein required for the stability of the *CLAVATA1* receptor-like kinase. Plant Cell 11: 1925–1934. 10521522 10.1105/tpc.11.10.1925 PMC144110

[62] Muller R, Bleckmann A, Simon R (2008) The receptor kinase CORYNE of *Arabidopsis* transmits the stem cell-limiting signal *CLAVATA3* independently of *CLAVATA1*. Plant Cell 209: 34–946. 10.1105/tpc.107.057547 PMC2390746 18381924

[63] Durbak AR, Taxt FE (2011) *CLAVATA* signaling pathway receptors of Arabidopsis regulate cell proliferation in fruit organ formation as well as in meristems. Genetics 189:177–194. 10.1534/genetics.111.130930 21705761 PMC3176120

[64] Bowman JL, Smyth DR (1999) *CRABS CLAW*, a gene that regulates carpel and nectary development in *Arabidopsis*, encodes a novel protein with zinc finger and helix-loop-helix domains. Development 126: 2387–2396. 10225998 10.1242/dev.126.11.2387

[65] Alvarez J, Smyth DR (1999) *CRABS CLAW* and *SPATULA*, two *Arabidopsis* genes that control carpel development in parallel with *AGAMOUS*. Development 126: 2377–2386. 10225997 10.1242/dev.126.11.2377

[66] Grabherr MG, Haas BJ, Yassour M, Levin JZ, Thompson DA, Amit I, et al (2011) Full-length transcriptome assembly from RNA-Seq data without a reference genome. Nat biotechnol 29: 644–652. 10.1038/nbt.1883 21572440 PMC3571712

[67] Mortazavi A, Williams BA, Mccue K, Schaeffer L, Wold B (2008) Mapping and quantifying mammalian transcriptomes by RNA-Seq. Nat Methods 5: 621–628. 10.1038/nmeth.1226 18516045 PMC13303166

