# Supplementary material for: Transcriptome Analysis of Flower Sex Differentiation in Jatropha curcas L. Using RNA Sequencing
Source: PLoS One. 2016 Feb 5;11(2):e0145613. doi: 10.1371/journal.pone.0145613 (PMC4746058; doi:10.1371/journal.pone.0145613)
Supplement: S1 File — Primers for the genes and reference gene used for the expression pattern analysis (Table B). (DOC) [file pone.0145613.s001.doc]

Table A The primers of the 12 selected unigenes and the reference gene (CL738.Contig3_S7) used for validation of expression trend.

| Genes | Primer |
| --- | --- |
| CL6457.Contig2_S7-F | TGAGCATAATGGCAGTGCCA |
| CL6457.Contig2_S7-R | TTGCTTTTATCCGGCCATCA |
| CL633.Contig1_S7-F | CTTGGAGACCCATCAGAGGCT |
| CL633.Contig1_S7-R | GTCAAGCCTGTCTGTGATGCC |
| Unigene26305 _S7-F | ACGAGGCTGTTCTTTGTGCA |
| Unigene 26305_S7-R | TTGCTTCCAGTGGACCTGAA |
| Unigene 8845 _S7-F | TAATGGCATCTGGCATGTGA |
| Unigene 8845 _S7-R | AGGCTCTTGGCTACGCTTTT |
| Unigene 169 _S7-F | AGGTTCAAAAGCCGGAATGC |
| Unigene 169 _S7-R | TTGCTTCAACCCTTTTGGCA |
| Unigene 25803 _S7-F | GCCATAATCCCCACTGGAAA |
| Unigene 25803 _S7-R | GACAAAACAATGGTGCGGTG |
| CL8050.Contig1_S7-F | GAACGAGCGACTAAGACAGCAA |
| CL8050.Contig1_S7-R | TTATCCAGAGTATGGCAACCCC |
| Unigene17081_S7-F | GACTAATAATGAGCAGATTTCGATGA |
| Unigene17081_S7-R | GGGGAAGACCGAGCGTAAG |
| Unigene24406_S7-F | GCTCAAGGGTGTTCTCGGC |
| Unigene24406_S7-R | ACTCAAGAACGAAACAGGAACCA |
| Unigene17053_S7-F | GGTTCTTGCGGTTGGGATT |
| Unigene17053_S7-R | TGTAGTGGAGGTCTGGTGCTGA |
| Unigene8655_S7-F | GCTTTTGGCTTCAACTGACTCTG |
| Unigene8655_S7-R | GATAGAAGGAGAAATGTGGGACCA |
| Unigene17829_S7-F | CTTCTTGGCATAAGTGGCATTCT |
| Unigene17829_S7-R | CTGGCAGGGAGGGAGATAGTTC |
| CL738.Contig3_S7(Tubulin-F) | CTCTGCAACTATGAGTGGTGTAACG |
| CL738.Contig3_S7(Tubulin-R) | CACGAGAAGTAAGTGGGGCAAA |

Table B The primers of the 16 selected unigenes and the reference gene (CL738.Contig3_S7) used for analysis their express pattern during the sexual differentiation process of flower.

| Genes | Primer |
| --- | --- |
| Unigene5429_S7-F | CCAAGTAGCCTTTCCCATTGATC |
| Unigene5429_S7-R | GGCTCGGGTAAGTGCGGT |
| Unigene5534_S7-F | CAAAGGTTGAAGGGTTCTGGAC |
| Unigene5534_S7-R | GCACCTTCCTACACCCAATCTT |
| CL3449.Contig2_S7-F | ACTCCTCTCCAACACAGGCTTC |
| CL3449.Contig2_S7-R | CCTGCTTGTGGCGTTCTCTAT |
| Unigene22184_S7-F | TCACGGAGGGAGAAGGAGAGA |
| Unigene22184_S7-R | TCCATACATTATCCTTGCGACC |
| Unigene19315_S7-F | TTCCAGACTCCATTTTCAGCCT |
| Unigene19315_S7-R | TTGGAGGCGAGGTAACGAAC |
| Unigene11972_S7-F | GGCACTCCATAAACGGTCAGG |
| Unigene11972_S7-R | TCTTCCCATAATGCCCCTCC |
| Unigene12973_S7-F | GAGGTTTGGGTTGTGGAGTATGAG |
| Unigene12973_S7-R | AGAGCAGTCTGTCCAAAAAGCG |
| Unigene4053_S7-F | TAGTGAGTGGGAAGAAGCCAATAGA |
| Unigene4053_S7-R | GCTAAACACGCTCTCTCTGCTCT |
| CL1944.Contig2_S7-F | GCTGGGGATTCAGTCCTTTTTA |
| CL1944.Contig2_S7-R | CAGTCTGAGGACGATTGGCAC |
| Unigene8983-F | TCAGAAATACACCCACTTACGCA |
| Unigene8983-R | CGAACTAACCGCTTTCCAATCT |
| Unigene3816_S7-F | AGGTTTCTTGATGCTGTTAGGTGTT |
| Unigene3816_S7-R | GGGTTTCTGAATCTTGAAGGGTT |
| Unigene25207_S7-F | AGAGGGAAGGCTACAATGGGTC |
| Unigene25207_S7-R | GCTTCTGAGCCTTTCATTATCCTC |
| CL8098.Contig1_S7-F | TAAAATACCCGAACACGACTGCT |
| CL8098.Contig1_S7-R | ATCCAACCATTTCTCCAAGCAC |
| Unigene11739_S7-F | ACTTGCTATCCTGAATCGCCTCT |
| Unigene11739_S7-R | AAACCCGACAACAATGAAGCC |
| Unigene2885_S7-F | GCGTCAAATAACACAATCCCAA |
| Unigene2885_S7-R | GAAGAAACCGAGAGCGAGAGAA |
| Unigene28_S7-F | GCACTCGCTTACCCTCAAACA |
| Unigene28_S7-R | TCACTCTAACGCAGCGAAATGT |
| CL738.Contig3_S7(Tubulin-F) | CTCTGCAACTATGAGTGGTGTAACG |
| CL738.Contig3_S7(Tubulin-R) | CACGAGAAGTAAGTGGGGCAAA |
