# Supplementary figures and images for: Transcriptome Analysis of Flower Sex Differentiation in Jatropha curcas L. Using RNA Sequencing
Source: PLoS One. 2016 Feb 5;11(2):e0145613. doi: 10.1371/journal.pone.0145613 (PMC4746058; doi:10.1371/journal.pone.0145613)

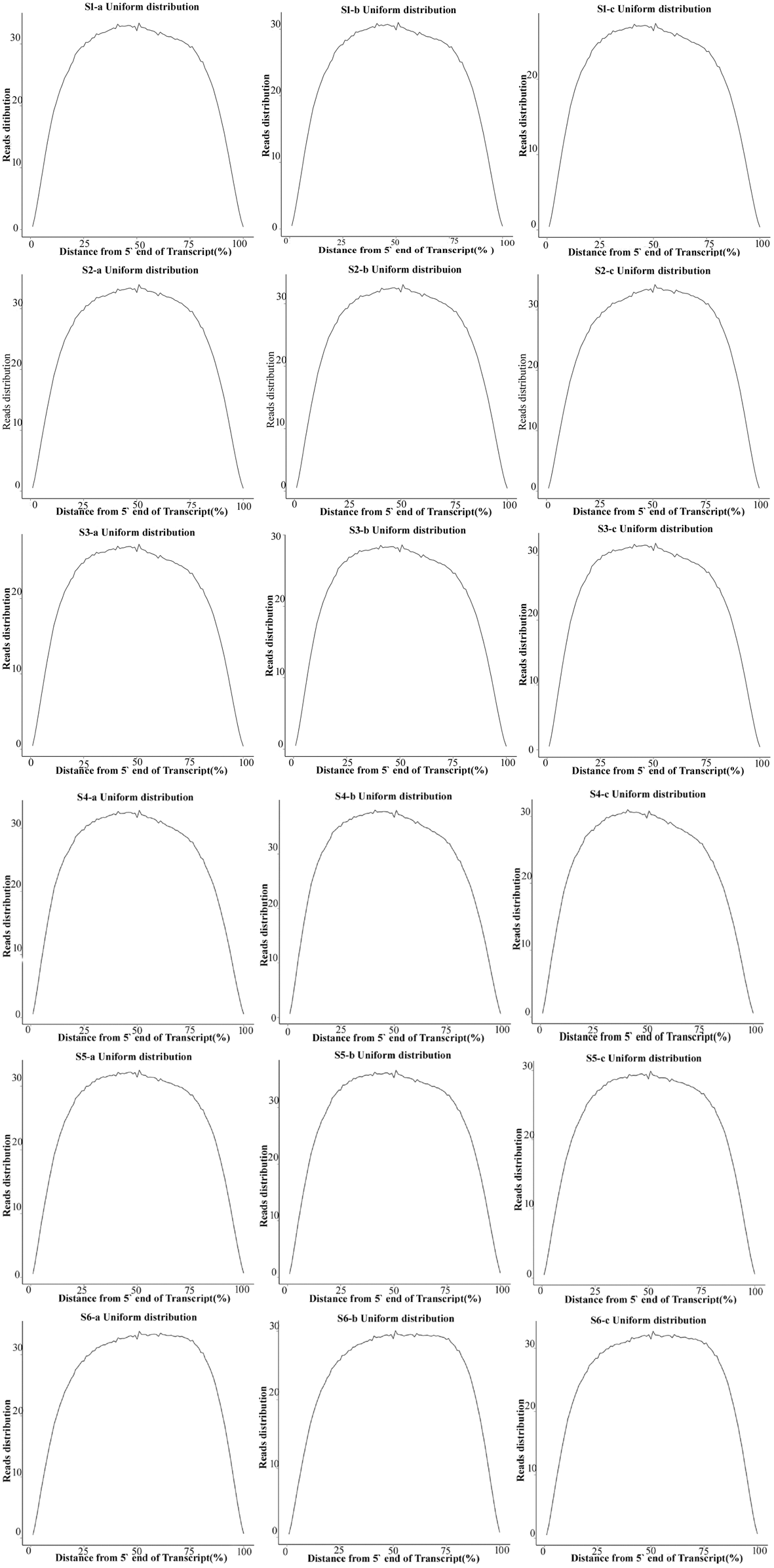

Supplement: S1 Fig — (TIF) [file pone.0145613.s002.tif]

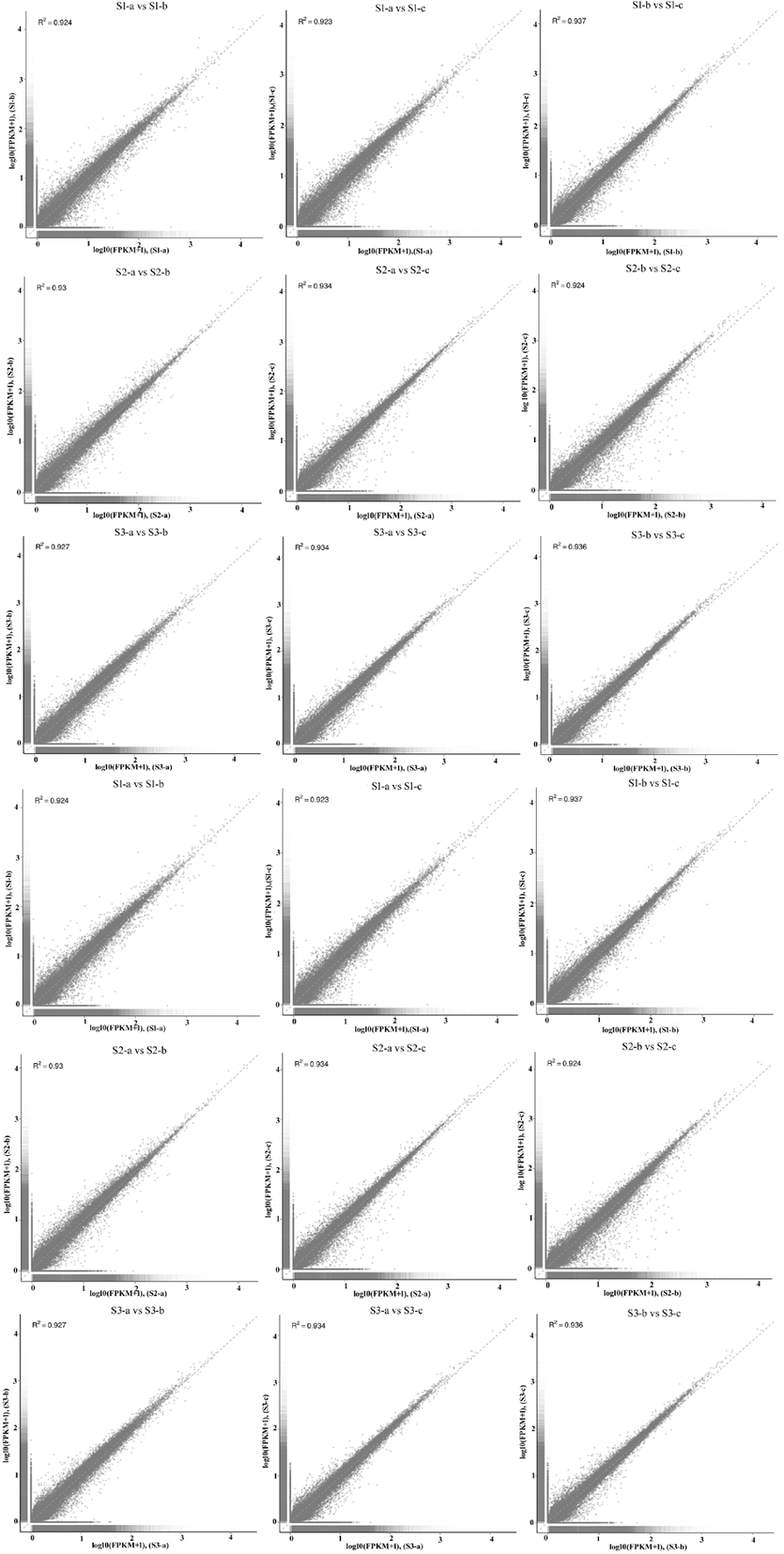

Supplement: S2 Fig — (TIF) [file pone.0145613.s003.tif]

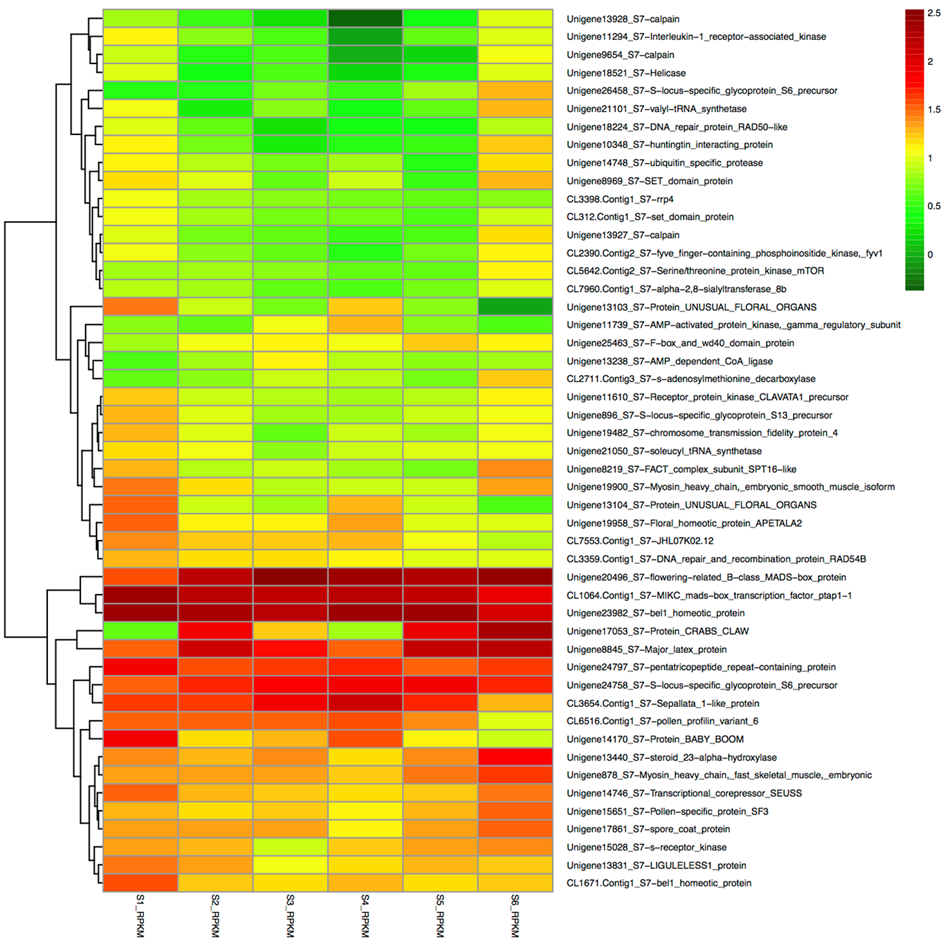

Supplement: S3 Fig — (TIF) [file pone.0145613.s004.tif]

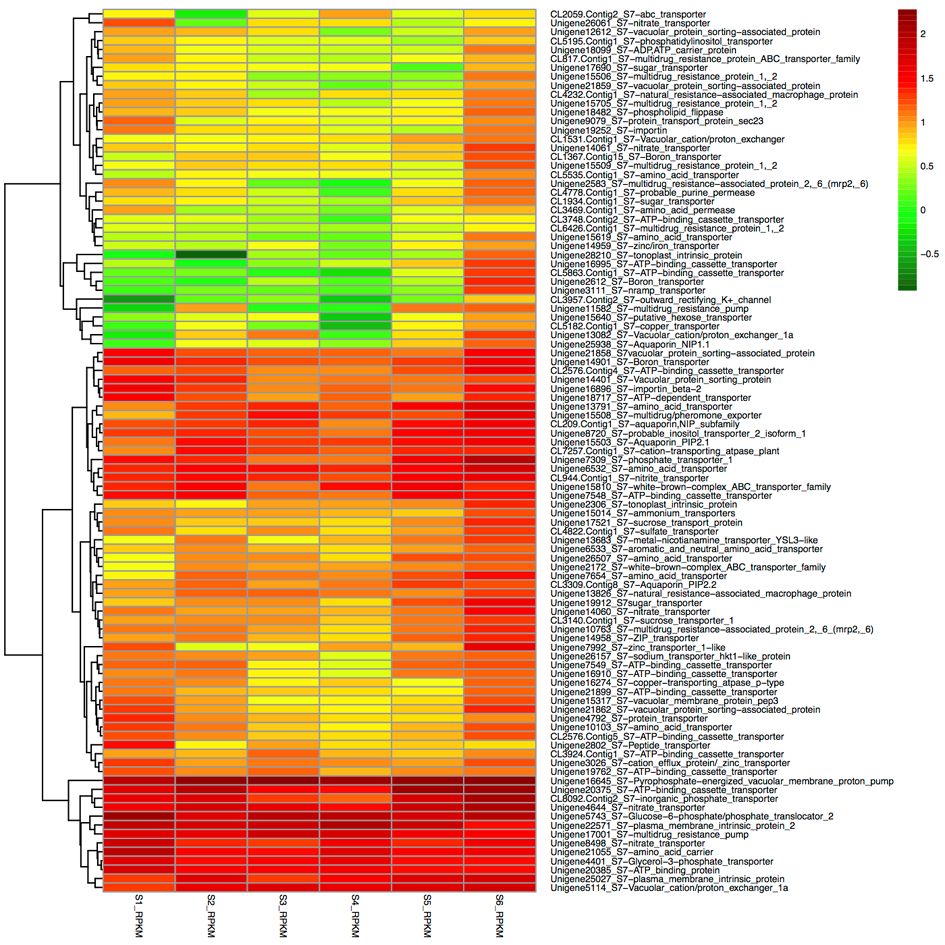

Supplement: S4 Fig — (TIF) [file pone.0145613.s005.tif]

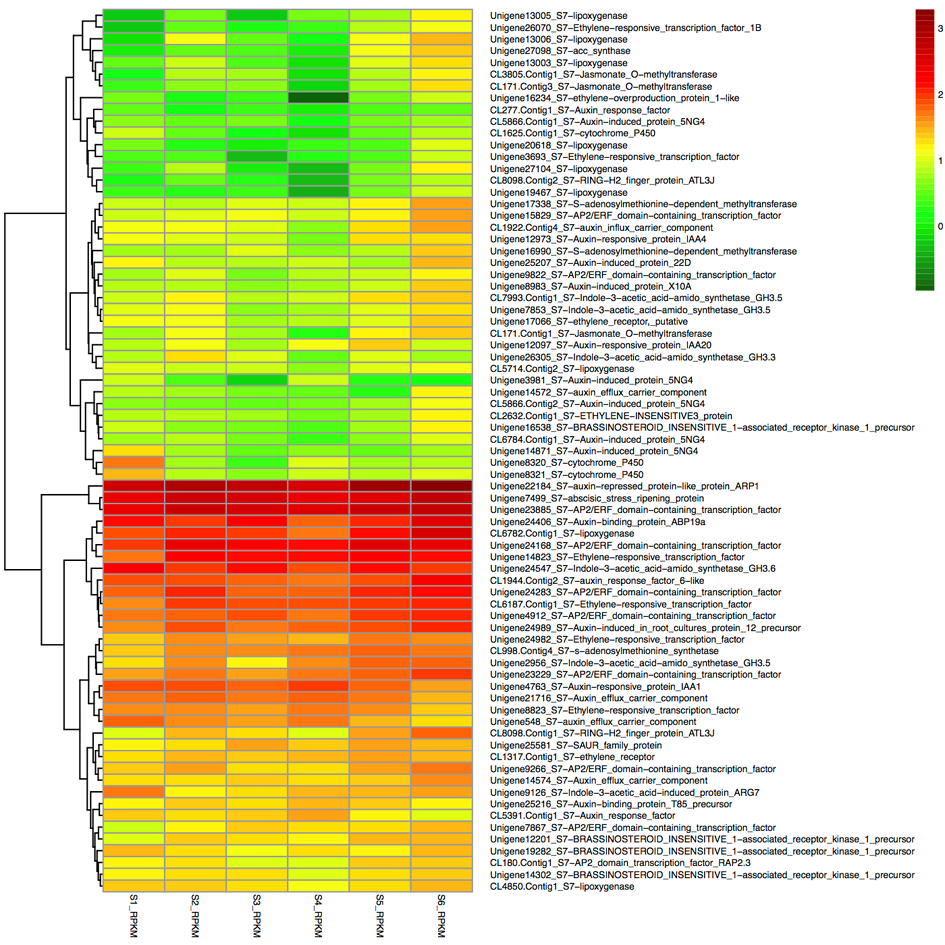

Supplement: S5 Fig — (TIF) [file pone.0145613.s006.tif]
